# Glucose, adrenaline and palmitate antagonistically regulate insulin and glucagon secretion in human pseudoislets

**DOI:** 10.1038/s41598-019-46545-6

**Published:** 2019-07-16

**Authors:** Estela Lorza-Gil, Felicia Gerst, Morgana Barroso Oquendo, Ulrich Deschl, Hans-Ulrich Häring, Mario Beilmann, Susanne Ullrich

**Affiliations:** 1 German Center for Diabetes Research (DZD e.V.), Tübingen, Germany; 2 Institute for Diabetes Research and Metabolic Diseases of the Helmholtz Zentrum München at the University of Tübingen (IDM), Tübingen, Germany; 3 University Hospital Tübingen, Internal Medicine IV, Endocrinology, Diabetology, Vascular Medicine, Nephrology and Clinical Chemistry, Tübingen, Germany; 4 Boehringer Ingelheim Pharma GmbH & Co. KG, Nonclinical Drug Safety, Biberach, Germany

**Keywords:** Gene expression analysis, Diabetes

## Abstract

Isolated human islets do not always meet the quality standards required for transplant survival and reliable functional *in vitro* studies. The formation of pseudoislets, i.e. the reaggregation of a defined number of islet cells after dissociation, improves insulin secretion. We present a simple method of pseudoislet formation from human islet cells and assess the transcriptome and function of isolated human islets and pseudoislets from the same organ donors. Following pseudoislet formation, insulin content/DNA and mRNA/RPS13 resembled that of islets. In pseudoislets, glucose-stimulated insulin secretion (GSIS) was significantly higher (8–13-fold) than in islets (2–4-fold). GSIS of pseudoislets was partly inhibited by the glucagon-like peptide-1 receptor (GLP-1R) antagonist exendin-9. The stimulatory effects of palmitate and forskolin at 12 mM glucose were also significantly higher in pseudoislets than in islets. Further analysis of pseudoislets revealed that regulation of secretion and insulin and glucagon content was maintained over a longer culture period (6–14 d). While adrenaline inhibited GSIS, adrenaline together with palmitate stimulated glucagon secretion 2-fold at low glucose, an effect suppressed by high glucose. Transcriptome analysis revealed that, unlike islets, pseudoislets were deprived of exocrine and endothelial cells. In conclusion, pseudoislet formation restores functional integrity of human islet cells and allows long-term *in vitro* testing.

## Introduction

Up to now, isolated human islets have been used for transplantation with only limited success^1^. Besides transplanted islets being subjected to immunological attacks, other undesired adverse effects also impact islet function^2,3^. Firstly, due to prolonged hypoxia, necrotic cell death occurs in the centre region of large isolated islets^4,5^. Secondly, following transplantation, a defective revascularization may hinder hormone dissemination^6^. Thirdly, the age and metabolic memory of human islets could have an impact on islet function after transplantation^7^. Jean-Claude Henquin’s careful evaluation of glucose-responsiveness of isolated human islets using a perifusion setting revealed no dependence of glucose-stimulated insulin secretion (GSIS) on donor age and islet size^8^. While an elevated body mass index (BMI) of the donor correlated with higher insulin content and secretion, the major adverse effect was the contamination of the islet preparation with exocrine tissue. Not only an incomplete purification of islets from exocrine tissue but also the dense packing of islets into a capsule for transplantation may affect islet function^9^. The use of reaggregated pseudoislets for transplantation was recently shown to result in improved glucose homeostasis^10^. Upon transplantation, revascularization remains defective in islets but is initiated in reaggregated pseudoislets^11^. Dissociated isolated islet cells are less glucose responsive than islet cells in clusters^12^. However, human islet cells exhibit improved glucose-responsiveness when attached to a matrix that enables them to spread^13^. The role of matrix and spreading for endocrine cell survival and function of human islet transplants has already been summarized elsewhere^14,15^. Another approach to improve glucose-responsiveness is the formation of pseudoislets^11^. In pseudoislets, hypoxia-damaged cells are eliminated and the superfusion with the stimulatory solution is ameliorated. Indeed, the limited oxygenation of the core of large islets favours hypoxia-induced apoptotic cell death and dysfunction^5,16^.

The fact that small islets display a better GSIS than large islets has been documented using isolated islets from rat and mouse^17–20^. Human islets vary also in their size and cell composition^21,22^. A higher percentage of glucagon-producing cells within the islet improves the glucose responsiveness^23,24^. By contrast, somatostatin exerts inhibitory effects on insulin and glucagon secretion^25,26^. A uniform distribution of α-, β- and δ-cells therefore reduces islet heterogeneity and variations for *in vitro* testing of islet function.

This study was initiated to evaluate the improvement of GSIS upon the formation of pseudoislets. This entailed comparing the glucose-responsiveness of the isolated human islets prior to dissociation with that of pseudoislets originating from the same organ donors. In addition, the transcriptomes of islets and pseudoislets were compared to determine whether differential expression underlies functional improvement. The results reveal a specific restitution of GSIS, together with a higher expression of β-cell markers, glucose transporter type 2 (GLUT-2), paired box gene 4 (PAX4) and islet-amyloid-polypeptide (IAPP) and a simultaneous reduction of acinar, ductal and endocrine cell markers. The pronounced inhibition of GSIS by adrenaline in the absence of endothelial cells suggests that adrenaline exerts a direct effect on β-cells and that insulin is released by regulated exocytosis. Furthermore, we ascertained that glucagon secretion is highest at low glucose in the presence of both palmitate and adrenaline, a stimulation inhibited by high glucose.

## Results

### Functional comparison of human islets and pseudoislets

**Figure 1:**
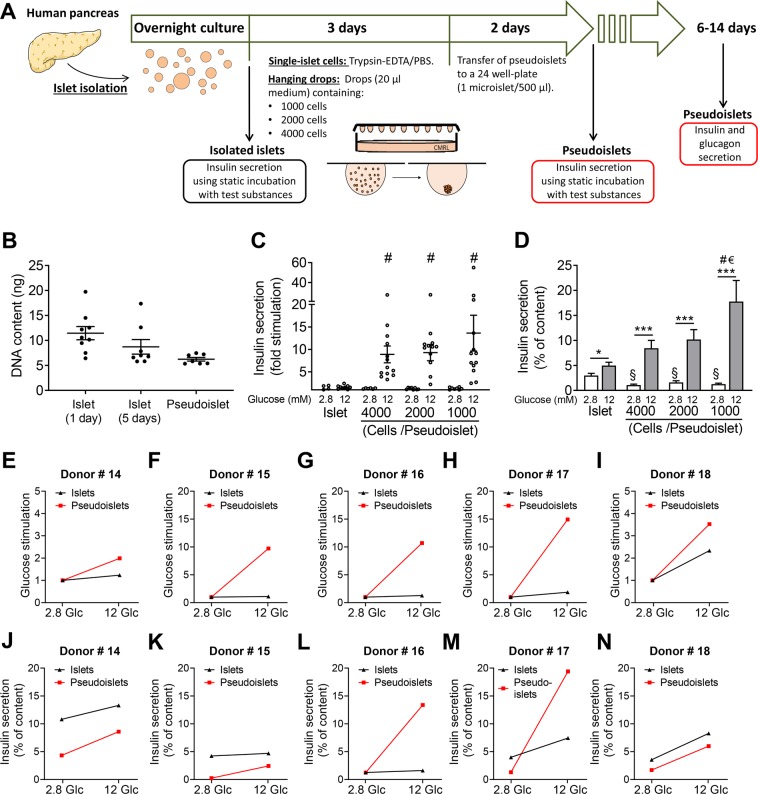
Reaggregation of human islet cells to pseudoislets specifically improves GSIS. **(A)** Experimental design of pseudoislet formation. Isolated human islets were cultured overnight. Non-dispersed islets were partly used for insulin secretion. The others (1000–2000 islets) were digested into single cells using trypsin. After digestion, a defined number of single cells were reaggregated in hanging drops of 20 µl over 3 days. After 2 days or longer culture in non-adhesive multi-well plates, the pseudoislets were used in the experiments. **(B)** DNA content of single islets and pseudoislets (formed from 2000 cells and cultured for 2 days) from the same donors (n = 2) were measured as described under Materials and Methods. **(C**,**D)** Comparison of GSIS of islets and pseudoislets reaggregated from 1000, 2000 and 4000 cells as indicated expressed as **(C)** fold-stimulation (12 mM glucose over 2.8 mM glucose). **(D)** % of insulin content (white bars, 2.8 mM glucose and grey bars, 12 mM glucose). Results are expressed as means + s.e.m. of n = 5 independent preparations. **(E–N)** GSIS of individual preparations of islets (black signs) and pseudoislets (red signs) expressed as **(E–I)** fold-stimulation and **(J–N)** % of insulin content. *p < 0.05, ***p < 0.0002 denotes significant GSIS; ^#^p < 0.05 denotes significant difference between secretion at 12 mM glucose of islets and the respective pseudoislet. ^§^p < 0.05 denotes significance to secretion of islets at 2.8 mM glucose. ^€^p < 0.05 significance between secretion at 12 mM glucose of 1000-cells and 4000-cells pseudoislets.

The ultimate functional test for β-cells is GSIS. Using a standard test of GSIS – static incubation for 1 h and increasing glucose concentrations – a high variation of both basal secretion and glucose responsiveness was observed in isolated human islets from non-diabetic organ donors (Suppl. Fig. 1A,B and Table 1). The mean glucose responsiveness did not become significant between 2.8 and 12 or 20 mM glucose (Suppl. Fig. 1C). In only 50% of the preparations did glucose augment insulin secretion more than 2-fold. Hypoxia prior to and during isolation and a prolonged shipping time is known to be detrimental to the islet core and function^4,5^. We therefore sought a method which improves islet cell function, ameliorates GSIS and allows long term testing. To this end, the human islets were dissociated into single cells and reaggregated to pseudoislets using the hanging drop method (Fig. 1A). To estimate the cell number, the DNA contents of islets and pseudoislets were measured (Fig. 1B). While the DNA content of islets was highly variable (5–20 ng), the DNA content of the pseudoislets was less variable (5–6 ng), reflecting a uniform pseudoislet size (Fig. 1B). The mean insulin content of islets was also very variable between the donors (Suppl. Fig. 1D). The insulin content per cell (i.e. DNA) varied by 20–40% between islets and pseudoislets of the same donor, indicating that pseudoislet formation did not affect insulin content of β-cells (Suppl. Fig. 1E,F). Pseudoislet formation initiated from 1000, 2000 or 4000 cells resulted in higher glucose responsiveness (8–13-fold stimulation) than non-dispersed islets cultured overnight (2-fold stimulation) from the same donor (Fig. 1C,D). This improvement in β-cell function is the result of lower basal secretion as well as of higher glucose-stimulation and was observed in every pseudoislet preparation (Fig. 1C–N). When non-dispersed isolated islets were cultured for the same length of time as the pseudoislets, the difference in GSIS remained significant (Suppl. Fig. 1G).

**Table 1:** Characteristics of islet donors.

Donor	Sex	Age (years)	BMI (kg/m^2^)	used for	Source
1	F	62	23.1	Suppl. Fig. 1A–D	ECIT
2	M	53	23.5	Suppl. Fig. 1A–D	ECIT
3	M	55	24.9	Suppl. Fig. 1A–D	ECIT
4	M	60	21.6	Suppl. Fig. 1A–D	ECIT
5	M	51	27.5	Suppl. Fig. 1A–D	ECIT
6	M	54	21.5	Suppl. Fig. 1A–D	ECIT
7	M	37	31.6	Fig. 2M; Suppl. Fig. 1A–D	ECIT
8	M	26	24.2	Fig. 2M; Suppl. Fig. 1A–D	ECIT
9	F	56	23	Suppl. Fig. 1A–D	ECIT
10	M	52	23.1	Suppl. Fig. 1A–D	ECIT
11	M	22	23	Fig. 2M; Suppl. Fig. 1A–D	ECIT
12	M	52	26.5	Suppl. Fig. 1A–D	ECIT
13	M	61	29.3	Suppl. Fig. 1A–D	ECIT
14	M	57	24.5	Figs 1B,C; 2K,L	ECIT
15	M	55	23.5	Figs 1B,C; 2K,L	ECIT
16	F	59	23.7	Figs 1B,C; 2K,L	ECIT
17	F	64	29	Figs 1B,C; 2K,L	ECIT
18	M	48	27.7	Figs 1B,C; 2K,L	ECIT
19	F	48	21.5	Fig. 4, Table 2, Suppl. Tables 1–2	Tebubio
20	M	62	24.5	Fig. 4, Table 2, Suppl. Tables 1–2	ECIT
21	F	43	34.1	Fig. 4, Table 2, Suppl. Tables 1–2	Tebubio
22	F	53	21.8	Fig. 4, Table 2, Suppl. Tables 1–2	ECIT
23	M	38	28	Fig. 2N	Tebubio
24	F	61	24.5	Fig. 2N	Tebubio
25	M	55	28.1	Fig. 2N	Tebubio
26	M	59	26.89	Fig. 3	InSphero
27	F	41	18.55	Fig. 3	InSphero
28	M	48	23.84	Fig. 3	InSphero
29	F	56	29.3	Fig. 3	InSphero
30	F	43	34.28	Fig. 3	InSphero
31	M	58	27.2	Fig. 1D; Suppl. Fig. 1E,F	ECIT
32	F	43	31.5	Fig. 1D; Suppl. Fig. 1E,F	Tebubio

**Figure 2:**
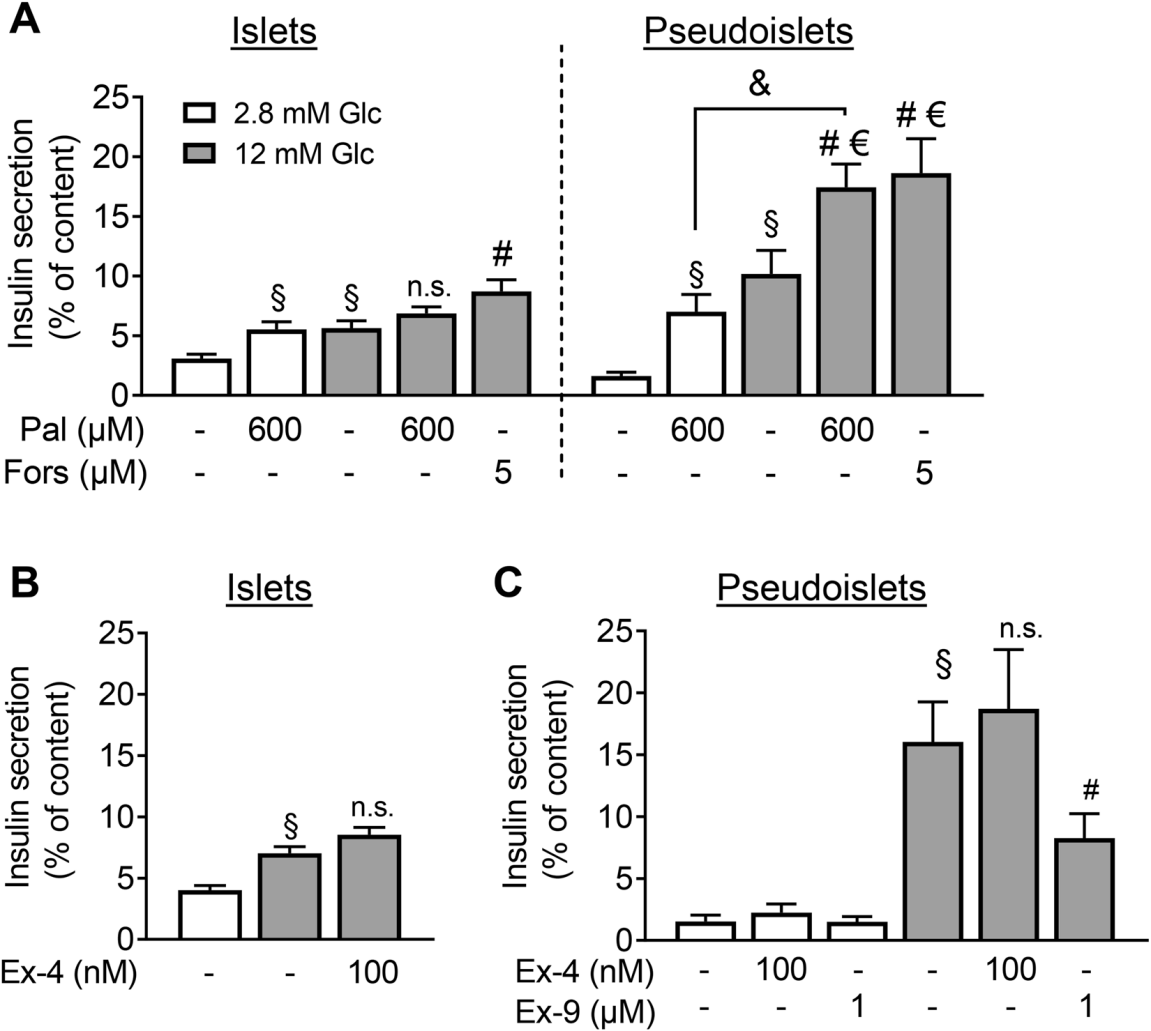
FFAR1 and GLP-1R mediated augmentation of GSIS. Isolated islets and pseudoislets (reaggregated from 2000 cells) were prepared, cultured for 2 days and incubated as described under Materials and Methods. Insulin secretion **(A**,**B)** of non-dispersed isolated islets (overnight culture) and **(A**,**C)** of the pseudoislets was stimulated as indicated. Results are expressed as means + s.e.m. of n = 3–5 independent preparations. ^§^p < 0.05 denotes significance to 2.8 mM glucose; ^#^p < 0.05 denotes significance to 12 mM glucose and ^&^p < 0.05 denotes significance between the two groups as indicated. ^€^p < 0.05 denotes significant difference between pseudoislets and islets at the same condition. White bars represent secretion at 2.8 mM glucose, grey bars at 12 mM glucose. Abbreviations used: Glc (glucose), Pal (palmitate), Fors (forskolin), Ex-4 (exendin-4), Ex-9 (exendin-9).

In addition to glucose, palmitate-stimulated insulin release was more efficient in pseudoislets than in islets at 12 mM glucose (€p < 0.05; Fig. 2A). At 2.8 mM glucose, palmitate augmented secretion in both islets and pseudoislets (Fig. 2A). Forskolin evoked a 2-fold enhancement of GSIS in islets and pseudoislets. Again, secretion was significantly higher in pseudoislets than in islets at 12 mM glucose (€p < 0.05, Fig. 2A). This effect was not mimicked by exendin-4 (Fig. 2B,C). However, the GLP-1R antagonist exendin-9 reduced GSIS by 50% in pseudoislets, suggesting that, under glucose stimulation, GLP-1R was already activated (Fig. 2C). These results indicate that pseudoislet formation improves GSIS which is partly antagonised by exendin-9, a GLP-1R inhibitor.

### Receptor effects after long term culture of pseudoislets: regulation of insulin and glucagon secretion by adrenaline

**Figure 3:**
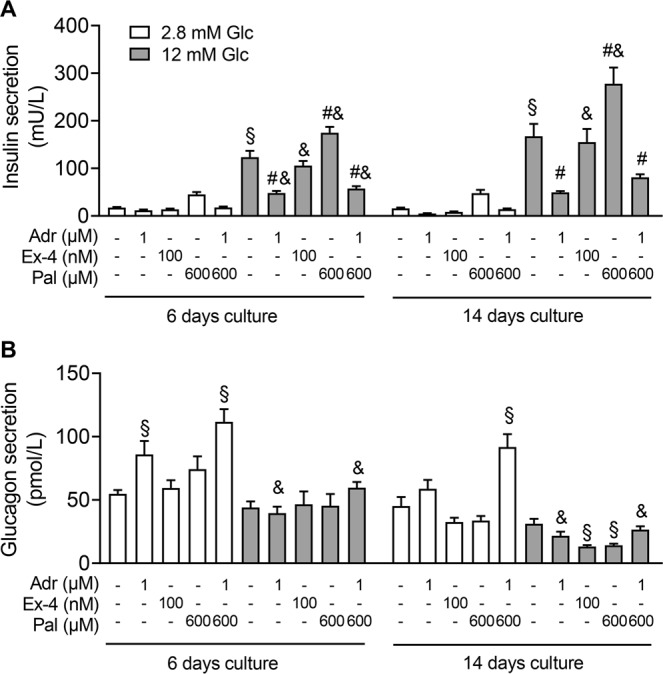
Long term functional integrity of pseudoislets: Opposing effects of glucose and adrenaline on insulin and glucagon secretion. Pseudoislets were cultured and incubated with test substances as described under Materials and Methods. (**A**) Insulin and (**B**) glucagon were measured in the same samples and are expressed as means + s.e.m. of n = 5 independent preparations. ^§^p < 0.05 denotes significance to 2.8 mM glucose, ^#^p < 0.05 significance to 12 mM glucose and ^&^p < 0.05 significance to 2.8 mM glucose of the respective group containing the same test substances. 2.8 mM glucose (white bars), 12 mM glucose (grey bars). Abbreviations used: Glc (glucose), Adr (adrenaline), Ex-4 (exendin-4), Pal (palmitate).

A stable functional integrity of pseudoislets over a period of weeks is a prerequisite for subchronic tests, such as toxicological testing. Insulin and glucagon content did not change during 8 days of culture (19.6 ± 2.9 ng and 19.8 ± 3.6 ng of insulin n = 5, 1.8 ± 0.3 ng and 1.5 ± 0.2 ng of glucagon n = 3 at 6 days and 14 days of culture, respectively). Glucose- and palmitate-dependent stimulation of insulin secretion was maintained in pseudoislets, even after an additional 8-days culture period under standard conditions (Fig. 3A). Secretion occurred in a regulated manner and receptors were functionally expressed in pseudoislets. This was endorsed by the pronounced inhibition of insulin secretion by adrenaline. Both, glucose- and palmitate-stimulated insulin secretion were significantly inhibited by adrenaline. Interestingly, adrenaline augmented glucagon secretion at low glucose, an effect which was more pronounced in the presence of palmitate (Fig. 3B). High glucose prevented the stimulatory effect of adrenaline on glucagon secretion. These results indicate that adrenaline is a potent regulator not only of insulin but also of glucagon secretion in human endocrine cells.

### Differential expression of β-cell specific genes in pseudoislets

**Figure 4:**
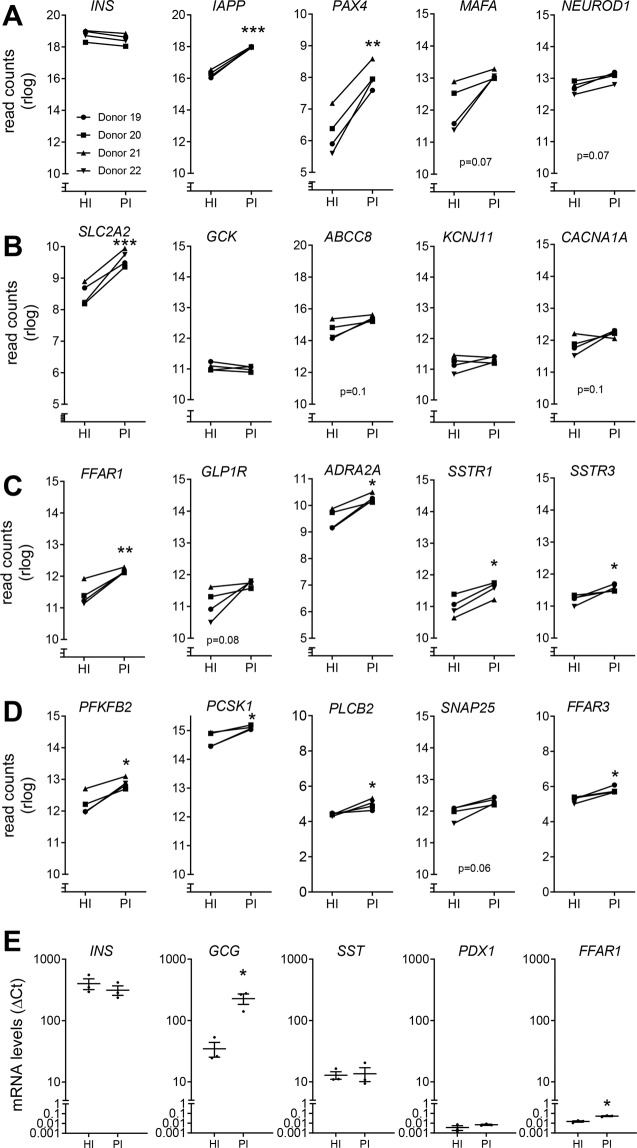
Transcriptome analyses revealed higher mRNA abundance of proteins expressed in β-cells in human pseudoislets than in isolated human islets from the same donors. (**A**–**D**) Islet (HI) and pseudoislet reaggregated from 2000 cells (PI) mRNA levels were measured by RNAseq as described under Materials and Methods. The data of 4 different donors are presented as log2 reads. (**E**) Relative mRNA levels of *INS*, *GCG*, *SST*, *PDX1 and FFAR1* in islets and pseudoislets were measured by RT-PCR. Asterisks denote significant differences between HI and PI, **(A–D)** padj < 0.05, R package; **(E)** t-test.

Transcriptome analyses of islets and pseudoislets were performed to determine the functional improvement observed in pseudoislets. This revealed a higher abundance of 198 mRNAs and a lower abundance of 367 mRNAs in pseudoislets than in islets from the same donors (2-fold change, Padj < 0.05; Suppl. Table 1). The comparatively high levels of insulin mRNA in islets (HI) and pseudoislets (PI) were confirmed by reverse transcriptase polymerase chain reaction (RT-PCR; Fig. 4A,E). The fact that islets and pseudoislets have similar amounts of insulin mRNA facilitates a comparison between the two with regard to the expression of other β-cell-specific genes. The mRNA levels of other β-cell-specific genes, in particular *IAPP* and *PAX4*, were significantly higher in pseudoislets than in islets, whereas mRNA levels of MAF BZIP transcription factor A (*MAFA)* and neuronal differentiation 1 (*NEUROD1)* remained unchanged (Fig. 4A). The augmented expression of GLUT-2 (*SLC2A2*), 6-phosphofructo-2-kinase/fructose-2,6-biphosphatase 2 *(PFKFB2*) and fatty acid receptor 1 (*FFAR1)* in pseudoislets could explain the increased responsiveness to glucose and palmitate, respectively (Fig. 4B–D). Other mRNA levels of proteins involved in GSIS such as glucokinase (*GCK)*, ATP binding cassette subfamily C member 8 (SUR1, *ABCC8*), potassium voltage gated channel subfamily J member 11 (*KCNJ11)* and calcium voltage-gated channel subunit alpha1A (*CACNA1A)* as well as *GLP1R* did not differ in the two preparations. In addition, relative mRNA levels of inhibitory receptors, such as the α_2_-adrenergic receptor (*ADRA2A*) and somatostatin receptors (*SSTR1* and *SSTR3*), were more abundant in pseudoislets than in islets (Fig. 4C). Other than higher mRNA levels of some of the enzymes involved in glucose metabolism, 2,6-phosphofructo-2-kinase/fructose-2,6-bisphosphatase (*PFKFB2*) and insulin processing enzyme proprotein convertase subtilisin/Kexin type 1 (*PCSK1*), mRNA levels of the other endocrine cell markers remained unchanged (Fig. 4 and Table 2).

**Table 2:** Transcriptome of human islets and pseudoislets: Note the higher levels of islet cell markers and the lower levels of extracellular matrix (ECM), apoptotic and inflammation markers in pseudoislets compared to islets. Endo, Endocrine; Aci, Acinar; Duct, Ductal; Endoth, Endothelial; PSCs, pancreatic stellate cells; MII/M, MHC class II and Mast cells (expressing cells according to ref.^39^).

	Protein	Gene name	Islet Mean	fold change	padj	Expressing cells					
						Endo	Exo	Duct	Endoth	PSCs	MII/M
Endocrine	Glucagon	GCG	1200392.9	1.91	0.103	+++	+	+	+	+	+
	Somatostatin	SST	81876.6	0.75	0.287	+++	+	+	+	+	+
	Pancreatic Polypeptide	PPY	6974.3	0.63	0.657	+++	+	+	+	+	+
	Proprotein Convertase Subtilisin/Kexin Type 2	PCSK2	27682.4	1.71	0.042	+++					
	Transmembrane 4 L6 Family Member 4	TM4SF4	21211.2	2.25	0.037	+++	+	+	+	+	
	Dipeptidyl Peptidase 4	DPP4	1825.4	2.42	4E-07	++		+			
	Calcium Voltage-Gated Channel Auxiliary Subunit Gamma 4	CACNG4	662.4	2.02	0.054	++					
	Protein Kinase C Gamma	PRKCG	117.6	2.09	0.025	+					
	Proteoglycan 4	PRG4	2762.1	0.37	0.012	++					
	Prostaglandin E Receptor 3	PTGER3	796.5	1.98	0.008	++					
ECM	CD93 molecule	CD93	1267.0	0.13	1E-10				+++		
	adenosine deaminase	ADA	39.2	0.39	0.002		+	+	++	+	
	atypical chemokine receptor 3	ACKR3	176.5	0.31	0.001		+	+	+		
	cadherin 5	CDH5	165.0	0.21	0.004				+++		
	calcium and integrin binding 1	CIB1	1781.0	0.50	0.009	+++	+++	+++	+++	+++	+++
	endothelial cell adhesion molecule	ESAM	284.6	0.24	2E-05			+	+++	+	
	intercellular adhesion molecule 1	ICAM1	581.2	0.42	0.044	+	++	++	+++		
	laminin subunit alpha 4	LAMA4	1323.2	0.44	0.002				+++	++	
	laminin subunit gamma 2	LAMC2	5127.7	0.27	0.008	+	++	+			
	myosin heavy chain 9	MYH9	20638.5	0.49	1E-04	++	+++	+++	+++	+++	
	platelet and endothelial cell adhesion molecule 1	PECAM1	1101.3	0.29	5E-04				+++		
	podocalyxin like	PODXL	1235.4	0.18	2E-10		+	+	+++		
	Podocalyxin Like 2	PODXL2	3460.1	2.15	8E-04	+++	+	+			
	protocadherin 12	PCDH12	172.7	0.45	2E-04				++		
	scavenger receptor class F member 1	SCARF1	152.4	0.39	1E-04				++		
	selectin E	SELE	62.2	0.08	5E-06				+		
	sidekick cell adhesion molecule 2	SDK2	411.2	0.48	1E-08	+					
	von Willebrand factor	VWF	660.6	0.23	0.003				++		
	Gap Junction Protein Alpha 1	GJA1	816.6	0.49	0.010		+	+	+++	+	
Inflammation	Interleukin 6	IL6	60.1	0.28	0.068					+	
	Macrophage Inflammatory Protein 1-Alpha	CCL3	15.5	0.30	0.004					++	+++
	Macrophage Inflammatory Protein 1-Beta	CCL4	29.4	0.37	0.023						+++
	Stromal Cell-Derived Factor 1 Receptor	CXCR4	371.3	0.23	0.061			+	+++		+
	Dendritic Cell And Monocyte Chemokine-Like Protein	CXCL17	68.1	0.13	0.009		+++	+		+	
	Interleukin 18 Binding Protein	IL18BP	237.7	0.43	0.005	+	+	+			
	Interleukin 1 Receptor Like 1	IL1RL1	167.9	0.03	0.005						+++
	Interleukin 32	IL32	713.0	0.30	0.012	+	+++	+++	+	+	+
	Interleukin 18 Receptor 1	IL18R1	73.8	0.42	0.081						++
	Interleukin 1 Receptor Type 2	IL1R2	67.8	0.30	0.116	+	+	+			
Apoptosis	DNA Damage Inducible Transcript 3	DDIT3	472.3	0.46	0.043	++	++	++	++	++	++
	DNA Damage Inducible Transcript 4	DDIT4	712.9	0.47	0.020	++	++	++	++	++	++
	G0/G1 Switch 2	G0S2	217.2	0.33	0.003	+	+	+		+	+
	GRAM Domain Containing 4	GRAMD4	2537.0	0.39	6E-05	+	+	+		+	+
	STEAP3 Metalloreductase	STEAP3	839.8	0.22	6E-08	+	++	+			+
	Calcium And Integrin Binding 1	CIB1	1781.0	0.50	0.009	+++	+++	+++	+++	+++	+++
	Egl-9 Family Hypoxia Inducible Factor 3	EGLN3	152.7	0.33	0.004	+	++	+			
	Endoplasmic Reticulum Oxidoreductase 1 Alpha	ERO1A	2895.1	0.49	0.029	+	+	+	+	+	+
	Endothelial Cell Surface Expressed Chemotaxis And Apoptosis Regulator	ECSCR	12.7	0.18	0.002				+++		
	Growth Arrest And DNA Damage Inducible Beta	GADD45B	860.8	0.28	0.002	+	+	+	+	+	+
	Serum/Glucocorticoid Regulated Kinase 1	SGK1	570.8	0.44	0.002	+	++	++	+++	+	+
	Transmembrane Protein 173	TMEM173	244.3	0.48	0.002	+	+		+++	++	+
	B Cell CLL/Lymphoma 6	BCL6	741.5	0.49	0.007	+	+	+		+	
	Bone Morphogenetic Protein 6	BMP6	33.4	0.22	1E-04	+			+		
	Early Growth Response 1	EGR1	1425.3	0.23	8E-04	++	++	++	++	++	++
	Roundabout Guidance Receptor 4	ROBO4	162.0	0.16	3E-07				+++		

Since insulin (*INS)*, glucagon (*GCG*) and somatostatin (*SST*) mRNAs are highly abundant, the mRNA levels were also estimated by RT-PCR (Fig. 4E). Interestingly, *GCG* mRNA levels were significantly higher in pseudoislets than in islets. The mRNA levels of proteins related to apoptosis and inflammation as well as the components of extracellular matrix (ECM) and endothelial cell markers were less abundant in pseudoislets than in islets (Table 2). As anticipated, pseudoislets were depleted of mRNAs of exocrine and ductal cell markers, indicative of the efficient purification of pseudoislets from contaminating exocrine tissue (Suppl. Table 2).

In conclusion, the pseudoislets prepared from adult human islets represent a purified and functional cell system suitable for long term *in vitro* studies.

## Discussion

Functional integrity of isolated human islets from organ donors is a major concern not only for clinical but also for scientific purposes. One problem of organ donors is the advanced age and organ fibrosis which have an impact on organ function. This study presents evidence that the preparation of pseudoislets allows selection of highly functional endocrine cells from organ donor islets. Most significantly, unlike for isolated islets, the regulation of insulin secretion was amended. In parallel, relative mRNA levels of β-cell markers were significantly higher in pseudoislets than in the originating islets. It is well known that hypoxia induce islet damage, particularly in the core of larger islets^4,5^. These damaged cells are removed upon dissociation and reaggregation of intact islet cells. Cell death may be one reason, why basal secretion is higher and GSIS lower in islets than in pseudoislets. Following isolation, human islets can often only be used after a prolonged shipping period, causing further damage to the islet preparation. Here, we present a simple method for the preparation of pseudoislets with functional integrity, even from human islets without proper GSIS.

Our results further suggest that pseudoislets reaggregated from 1000-cells respond better to glucose than pseudoislets from 4000-cells (Fig. 1D). It has already been observed that smaller islets have a functional superiority over larger ones^18^. Interestingly, the size of neighbouring islets within the human pancreas is highly variable and small islets prevail. However, the overall β-cell mass is generally determined by large islets^20,27^. Of note, the isolation procedure is selective for bigger islets, while small islets are often digested and therefore lost. We therefore speculate that *in vivo* within the pancreatic tissue the small islets are relevant for islet function, whereas large islets predominantly account for β-cell mass. New data suggest that early stages in the development of diabetes are accompanied by dysfunctional hormone secretion leading to hyperglycemic episodes, albeit not by a decline of β-cell mass^11^. This observation attaches only minor importance to the reduction of β-cell mass in the development of type 2 diabetes mellitus (T2DM).

The preparation of pseudoislets not only enables us to generate spheroids identical in size and functional response and, therefore, useful for *in vitro* studies, but also to control the composition of pseudoislets, i.e. the ratio of β-:α-:δ-cells^28^ and the addition of other cell types such as endothelial cells, smooth muscle cells, precursor cells and components of the extracellular matrix. A standardized functional test, i.e. GSIS, is both simple and sensitive on account of its dependence on intact metabolism. In pseudoislets, GSIS and insulin content remain stable over 8-days cultures. This renders them useful for long-term toxicological studies, e.g. for the identification of drug-induced dysfunctions and structural alterations of native pancreatic endocrine cells. The pseudoislet preparation may also be suitable to examine the capacity of human β-cell to synthesize sufficient insulin upon repeated stimulation. The use of human pseudoislets has therefore an advantage over the use of isolated native human islets which is hampered by a highly variable cell composition and necrotic islet core, and also over cell lines which have an unphysiological high proliferation rate.

In contrast to published observations, the activation of GLP1R by exendin-4 did not enhance GSIS in islets and pseudoislets^29^. Interestingly, in pseudoislets improved GSIS was inhibited by 50% by exendin-9, an antagonist of GLP-1R. This observation suggests that GLP-1R signalling is already activated in pseudoislets. The importance of GLP-1R for GSIS has already been demonstrated with mouse islets^30^. Using *in situ* perfusion of mouse pancreas, proper GSIS was shown to depend on the paracrine effects of glucagon which are exerted not only through glucagon receptor but also through GLP-1R^31^. Here, our data suggest that a similar mechanism may apply to human islet cell interactions, and that glucagon released from α-cells activated β-cell GLP-1R and contributes to the improvement of GSIS in pseudoislets compared to islets. In humans, the main site of action of GLP-1 that improves glucose homeostasis is not completely understood^32^. The concentration of GLP-1 that augments GSIS *in vitro* is much higher than the concentration in blood. In contrast to exendin-4, forskolin augmented GSIS 2-fold in both preparations. The direct stimulation of adenylyl cyclase by forskolin leads firstly to an excessive augmentation of cellular cAMP levels and, secondly, bypasses receptor regulation of adenylyl cyclase^33^. Not only is the enzyme activity stimulated by a variety of different receptors, it is also potently inhibited through receptors present in β-cells, such as adrenoceptors and somatostatin receptors^34^.

The direct inhibitory effect of adrenaline on insulin secretion has been well studied in rodents^35,36^. In humans, an inhibitory action through α_2_-adrenoceptor (*ADRA2*) activation has been documented, although sympathetic nerve endings have been found only in association with endothelial cells^37,38^. Of note, the receptor is activated both by sympathetic release of noradrenaline and by humoral action of adrenaline. Our RNAseq data suggest that *ADRA2A* is the main receptor subtype expressed in human islet cells. *ADRA2C* and *ADRA2B* mRNA levels were much lower than *ADRA2A* (baseMeans were 1030 (DESeq2 normalized counts) for *ADRA2A*, 2.5 for *ADRA2B*, 45 for *ADRA2C)*. Single islet cell analysis suggests that *ADRA2A* is expressed mainly in β-cells^39^.

Adrenaline not only suppressed GSIS but also increased glucagon secretion at low, 2.8 mM glucose, an effect accentuated by palmitate. The stimulatory effect of adrenaline on α-cells has already been described using mouse islets and purified rat α-cells^40–42^. The levels of fatty acids and adrenaline in blood are at their highest during fasting and stress, conditions dominated by glucagon-induced mobilisation of glucose^43^. Beta-1 adrenergic receptor (*ADRB1)* mRNA was found exclusively in α-cells, while *ADRB2* in α- as well as in a number of other endocrine islet cells^39^. *FFAR1* is expressed in β- and α-cells. Our results suggest that adrenaline is an important regulator of glucagon secretion and that its stimulatory effect is inhibited by high glucose. The effect of glucose on glucagon release seems to be insulin independent since insulin secretion is suppressed in the presence of adrenaline (Fig. 3A,B, compare the last two columns).

Islet transplantation is mainly required for type 1 diabetes mellitus (T1DM) therapy^1^. The major concern with regard to the transplanted islets is the autoimmune destruction of β-cells. A certain degree of protection of transplanted islets is effected by encapsulation of the transplant^9^. Such an approach, however, leads to a tight packing of islets which, in turn, may not be conducive to islet function and survival. Revascularization, an approach with which transplant survival can be improved^44^, was recently observed to be suppressed in intact islets due to vessel debris but improved in reaggregated small pseudoislets^6^. In this sense, small reaggregates survive thanks to a better oxygenation and perfusion but also due to a better revascularization prognosis.

In conclusion, using pseudoislets reaggregated from isolated human islet cells, we show a GLP1R-dependent improvement of GSIS and identify adrenaline not only as a potent inhibitor of GSIS but also as an activator of glucagon secretion. Pseudoislets are suitable for functional studies of native human endocrine cells. They are a promising tool for toxicological studies to investigate drug-induced functional and structural adverse effects and may also play an important role in future transplantations.

## Materials and Methods

### Human islet cell isolation and pseudoislet preparation

The human islets used in this study were procured through the European Consortium for Islet Transplantation (ECIT Centers in Geneva, Lille and Milano, JDRF award 31-2008-416 for basic research program) or purchased from Tebu-Bio (Offenbach, Germany). The donor characteristics are provided in Table 1. Donors gave informed consent for the use of their islets preparations in scientific research. Ethical approval for the use and for the procedures and protocols involved in handling of human islets isolated from organ donors was obtained from the Ethics Commission of the Medical Faculty of the Eberhard-Karls-University and the University Hospital of Tübingen (533/2010BO2 and 098/2017BO1). All experiments and methods involving human islets and islet cell aggregates were performed in accordance with the above mentioned approvals, guidelines and regulations.

To reduce exocrine contamination, the islets were cleaned immediately upon arrival by hand picking under a stereo dissecting microscope in Hank’s balanced salt solution supplemented by 0.25% bovine serum albumin (BSA). After an overnight culture in CMRL1066 medium containing (in mM): 5 glucose, 10 Hepes, 2 L-glutamine supplemented with 10% (v/v) fetal calf serum (FCS, Serva, Heidelberg, Germany) and antibiotics (100 U/ml), the islets were dissociated into single cells using trypsin (42.5 U/mL) in phosphate buffered saline containing 2% (w/v) ethylenediaminetetraacetic acid (EDTA-PBS) and gentle shaking. Following dissociation, cell viability (>95%) was assessed by trypan blue staining. Drops (20 µl of medium) containing 1000, 2000 or 4000 cells were placed onto the top of a petri dish and cultured as hanging drops for 3 days (Fig. 1). Medium was added to the bottom of the petri dishes to avoid drying out. The cells reaggregated within the hanging drop to form one pseudoislet. Each pseudoislet was then placed into a non-adhesive well of a 24-well-plate containing 0.5 ml medium and cultured for another 2 days. Pseudoislets purchased from InSphero (InSphero AG, Schlieren, Switzerland) were cultured for 6 days and 14 days before functional testing (Fig. 3).

### Measurement of insulin and glucagon secretion

Isolated islets and pseudoislets were tested in parallel for insulin secretion. The islets or pseudoislets were carefully washed and preincubated for 1 h in modified Krebs-Ringer buffer (KRB) containing (in mM): 135 NaCl, 4.8 KCl, 2.6 CaCl_2_, 1.17 KH_2_PO_4_, 1.18 MgSO_4_, 5 NaHCO_3_, 10 HEPES, 2.8 glucose and 0.5% BSA.

Thereafter, isolated islets (5 islets/0.5 ml) and pseudoislets (1 pseudoislet/0.1 ml) were incubated for 1 h in fresh KRB containing test substances at appropriate concentrations. Secreted insulin or glucagon in the supernatant and insulin or glucagon content after extraction in acid ethanol (1.5% (v/v) HCl in 75% (v/v) ethanol) was measured using a radioimmunoassay (Millipore, USA) or a sensitive enzyme-linked immunosorbent assay (ELISA; Mercodia, Sweden).

### DNA measurements

DNA of samples containing 10 islets or 10 pseudoislets was extracted using QIAamp DNA-Mico Kit (Qiagen, Hilden, Germany) in accordance with the manufacturer’s instructions. DNA concentration was measured by fluorescence using the Qubit® dsDNA HS Assay Kit and Qubit® 3.0 Fluorometer (Thermo Fisher Scientific, Waltham, U.S.).

### Transcriptome profiling

Aliquots of cultured human islets and their respective pseudoislets were used for RNA extraction using NucleoSpin® RNA XS (Macherey-Nagel, Düren, Germany). RNA integrity (RIN ≥ 9) was measured with Bioanalyzer 2100 (Agilent Technologies). Sequencing was performed as described previously at the Center for Molecular and Cellular Bioengineering (Andreas Dahl, CMCB, Technical University Dresden, Germany)^45^. In short, mRNA was isolated from 120 ng RNA by poly-dT enrichment using the NEBNext Poly(A) mRNA Magnetic Isolation Module. After fragmentation, the samples were subjected to the workflow for strand-specific RNASeq library preparation (Ultra Directional RNA Library Prep II, NEB) and 75 bp single read sequencing was performed on Illumina NextSeq500. After sequencing, FastQC was used to perform quality control. Differential expression between islets and pseudoislets was tested with DESeq R package (v.1.10.1). The complete analysis is available in Supplementary File 2 and under GEO code GSE133903.

### RT-PCR

RNA (100 ng) was transcribed using a recombinant reverse transcriptase (Transcriptor first strand cDNA synthesis kit #4897030001, Roche Diagnostics, Rotkreuz, Switzerland). PCR was performed with the LightCycler480 Probes Master system (Roche Diagnostics GmbH, Mannheim, Germany). Specific primers used were: *hINS2* up: 5′-AGGCTTCTTCTACACACCCAAG-3′, down: 5′-CACAATGCCACGCTTCTG-3′, probe #27; for *hGCG* up: 5′-TCTGTTCTACAGCACACTACCAGA-3′, down: 5′-AGCTTGCCTTGTACCAGCATT-3′, probe #5; for *hSST* up: 5′-ACCCCAGACTCCGTCAGTTT-3′, down: 5′- ACAGCAGCTCTGCCAAGAAG-3′, probe #38; for *hPDX1* up: 5′-AAGCTCACGCGTGGAAAG-3′, down: 5′-GCCGTGAGATGTACTTGTTGAA-3′, probe #78; for *hFFAR1* up: 5′-GTGTCACCTGGGTCTGGTCT-3′, down: 5′-CCAGGGAGGTGTTGCTGT-3′, probe #20. The housekeeping gene used was *hRPS13* up: 5′-GGTTGAAGTTGACATCTGACGA-3′, down: 5′-CTTGTGCAACACCATGTGAA-3′, probe #68. Gene expression was quantified by the 2^−(ΔCt)^ method relative to the housekeeping gene.

### Statistics

Statistical analysis of insulin and glucagon secretion was performed using GraphPad Prism (GraphPad Software Inc, La Jolla, CA). One way ANOVA and Tukey post testing was used when more than 2 conditions were compared. For glucose stimulation (GSIS) Student’s t-test for unpaired two samples was used and is marked by *. Differences were considered statistically significant at p ≤ 0.05. RNAseq data were analysed using DESeq R package (v.1.10.1).

## Supplementary information

Data set 1

Data set 2
