# Supplementary material for: Glucose, adrenaline and palmitate antagonistically regulate insulin and glucagon secretion in human pseudoislets
Source: Sci Rep. 2019 Jul 16;9:10261. doi: 10.1038/s41598-019-46545-6 (PMC6635387; doi:10.1038/s41598-019-46545-6)
Supplement: Supplementary file 1 — Data set 1 [file 41598_2019_46545_MOESM1_ESM.pdf]

Glucose, adrenaline and palmitate antagonistically regulate insulin and glucagon secretion in human pseudoislets

Estela Lorza-Gil, Felicia Gerst, Morgana Barroso Oquendo, Ulrich Deschl, Hans-Ulrich Häring, Mario Beilmann* and Susanne Ullrich*

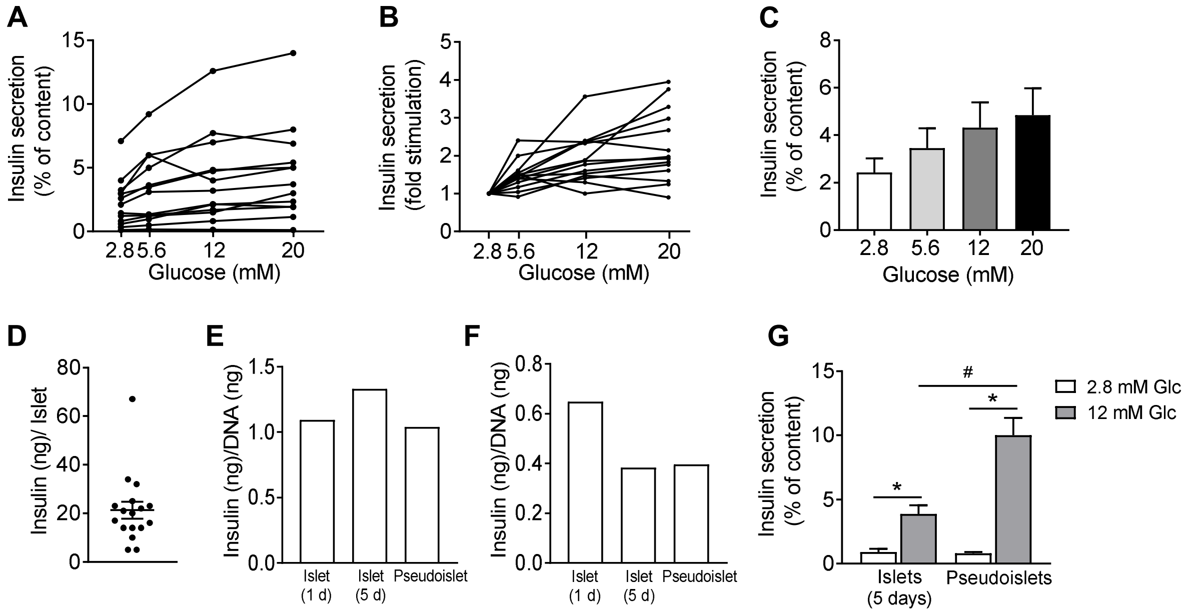

Supplementary Figure 1 Glucose-stimulated insulin secretion of isolated human islets. Human islets were cultured and GSIS measured as described under Materials and Methods. **(A-C)** GSIS of 13 human islet preparations of different donors expressed as **(A)** % of insulin content and **(B)** glucose stimulation (fold increase over 2.8 mM glucose). **(C)** Data of (A) expressed as means + s.e.m. **(D)** Mean islet insulin content of 13 preparations. **(E, F)** Comparison of insulin content/DNA of islets and pseudoislets after 1 d and 5 d of culture as indicated from 2 donors (E donor#31, F donor#32) **(G)** Comparison of GSIS of islets and pseudoislets, both cultured for 5 d. Results are expressed as means + s.e.m. of n = 8 observations from 2 preparations. 2.8 mM glucose (white bars), 12 mM glucose (grey bars), * denotes significant GSIS, # denotes significant different secretion at 12 mM glucose between islets and pseudoislets.

Supplementary Table 1 Transcriptome analysis of human islets and pseudoislets by RNAseq. All genes differentially expressed (>2-fold, padj<0.05) in pseudoislets compared to islets. The mRNA levels of 198 genes were higher (>2-fold) and 367 genes were reduced (<2-fold) in pseudoislets when compared to islets (padj<0.05).

A) Differentially expressed genes higher in pseudoislets when compared to islets (>2-fold, padj<0.05).

Gene stable ID	Gene name	baseMean	log2FoldChange	lfcSE	stat	pvalue	padj
ENSG00000121351	IAPP	192670.24	2.49	0.19	13.31	1.90E-40	3.50E-36
ENSG00000169903	TM4SF4	21211.24	1.17	0.39	3.01	2.64E-03	3.72E-02
ENSG00000167615	LENG8	7520.34	1.18	0.29	4.05	5.07E-05	2.30E-03
ENSG00000131094	C1QL1	4837.17	1.13	0.36	3.11	1.85E-03	3.02E-02
ENSG00000075035	WSCD2	4296.33	1.39	0.31	4.53	6.01E-06	4.63E-04
ENSG00000126266	FFAR1	3845.74	1.03	0.26	3.99	6.51E-05	2.81E-03
ENSG00000166923	GREM1	3758.20	1.54	0.44	3.49	4.87E-04	1.20E-02
ENSG00000114631	PODXL2	3460.07	1.11	0.25	4.37	1.22E-05	7.87E-04
ENSG00000117472	TSPAN1	2887.17	1.56	0.20	7.97	1.56E-15	5.68E-12
ENSG00000175262	C1orf127	2885.55	1.09	0.34	3.19	1.42E-03	2.53E-02
ENSG00000136883	KIF12	2748.66	1.04	0.36	2.91	3.66E-03	4.56E-02
ENSG00000174871	CNIH2	2291.28	1.29	0.18	6.99	2.84E-12	2.16E-09
ENSG00000139329	LUM	2235.16	1.06	0.36	2.93	3.43E-03	4.37E-02
ENSG00000140465	CYP1A1	1968.17	1.66	0.52	3.20	1.36E-03	2.46E-02
ENSG00000197635	DPP4	1825.37	1.28	0.21	6.08	1.17E-09	4.46E-07
ENSG00000197119	SLC25A29	1450.82	1.10	0.16	7.04	1.90E-12	1.65E-09
ENSG00000019582	CD74	1353.56	1.14	0.32	3.57	3.56E-04	9.57E-03
ENSG00000150594	ADRA2A	1030.25	1.11	0.29	3.86	1.15E-04	4.26E-03
ENSG00000165899	OTOGL	1009.17	1.11	0.33	3.36	7.72E-04	1.69E-02
ENSG00000145362	ANK2	974.63	1.09	0.35	3.10	1.94E-03	3.11E-02
ENSG00000171551	ECEL1	940.92	1.41	0.34	4.15	3.29E-05	1.68E-03
ENSG00000173805	HAP1	773.35	1.61	0.29	5.47	4.56E-08	9.15E-06
ENSG00000153012	LGI2	737.90	1.36	0.34	3.95	7.73E-05	3.21E-03
ENSG00000049089	COL9A2	722.73	1.21	0.29	4.20	2.67E-05	1.43E-03
ENSG00000153822	KCNJ16	721.14	1.48	0.33	4.54	5.57E-06	4.34E-04
ENSG00000163581	SLC2A2	636.55	1.67	0.30	5.53	3.18E-08	6.53E-06
ENSG00000123243	ITIH5	602.57	1.21	0.36	3.34	8.47E-04	1.79E-02
ENSG00000168309	FAM107A	599.79	1.27	0.37	3.42	6.28E-04	1.47E-02
ENSG00000144152	FBLN7	598.79	1.20	0.19	6.36	1.96E-10	9.95E-08
ENSG00000143630	HCN3	594.79	1.34	0.25	5.46	4.82E-08	9.56E-06
ENSG00000196169	KIF19	562.21	1.10	0.37	2.99	2.76E-03	3.82E-02
ENSG00000134827	TCN1	489.76	3.79	0.80	4.76	1.93E-06	1.79E-04
ENSG00000182752	PAPPA	474.60	1.12	0.28	4.04	5.34E-05	2.40E-03
ENSG00000170099	SERPINA6	473.17	1.89	0.56	3.37	7.49E-04	1.65E-02
ENSG00000152953	STK32B	446.91	1.07	0.36	3.01	2.64E-03	3.72E-02
ENSG00000099869	IGF2-AS	440.59	1.56	0.53	2.91	3.56E-03	4.50E-02
ENSG00000113924	HGD	439.67	1.03	0.28	3.63	2.83E-04	8.23E-03
ENSG00000174358	SLC6A19	437.83	1.22	0.40	3.02	2.54E-03	3.63E-02
ENSG00000119943	PYROXD2	431.09	1.37	0.43	3.16	1.59E-03	2.72E-02
ENSG00000116032	GRIN3B	425.79	2.77	0.55	5.06	4.14E-07	5.31E-05
ENSG00000112276	BVES	388.89	1.20	0.36	3.30	9.57E-04	1.95E-02
ENSG00000174827	PDZK1	372.83	1.46	0.47	3.12	1.78E-03	2.95E-02
ENSG00000182575	NXPH3	369.49	1.06	0.29	3.64	2.75E-04	8.07E-03
ENSG00000163462	TRIM46	359.52	1.04	0.24	4.42	9.75E-06	6.72E-04
ENSG00000175868	CALCB	334.17	1.76	0.52	3.39	6.96E-04	1.57E-02
ENSG00000070601	FRMPD1	331.07	1.32	0.42	3.12	1.79E-03	2.96E-02
ENSG00000064300	NGFR	321.25	3.12	0.86	3.63	2.82E-04	8.20E-03
ENSG00000084453	SLCO1A2	287.69	1.18	0.21	5.57	2.60E-08	5.52E-06
ENSG00000166959	MS4A8	273.17	1.01	0.22	4.51	6.46E-06	4.91E-04
ENSG00000125492	BARHL1	265.73	1.97	0.37	5.27	1.39E-07	2.18E-05
ENSG00000143341	HMCN1	238.30	1.59	0.43	3.66	2.56E-04	7.67E-03
ENSG00000124491	F13A1	237.83	1.45	0.44	3.33	8.68E-04	1.81E-02
ENSG00000259834	AL365361.1	231.48	1.06	0.22	4.78	1.75E-06	1.67E-04
ENSG00000241388	HNF1A-AS1	223.98	1.48	0.34	4.30	1.73E-05	1.02E-03
ENSG00000106331	PAX4	214.31	2.61	0.61	4.28	1.91E-05	1.10E-03
ENSG00000230006	ANKRD36BP2	206.54	1.20	0.29	4.17	2.99E-05	1.57E-03
ENSG00000121207	LRAT	205.87	2.04	0.48	4.29	1.82E-05	1.06E-03
ENSG00000230928	AL139241.1	197.60	1.77	0.56	3.14	1.68E-03	2.82E-02
ENSG00000183379	SYNDIG1L	195.43	2.47	0.65	3.82	1.31E-04	4.70E-03
ENSG00000184221	OLIG1	195.31	1.12	0.36	3.10	1.93E-03	3.11E-02
ENSG00000135472	FAIM2	188.46	1.79	0.30	5.99	2.07E-09	7.55E-07
ENSG00000103485	QPRT	180.31	1.14	0.26	4.40	1.09E-05	7.27E-04
ENSG00000206120	EGFEM1P	176.47	1.65	0.31	5.40	6.74E-08	1.24E-05
ENSG00000108932	SLC16A6	156.76	1.20	0.28	4.25	2.14E-05	1.20E-03
ENSG00000134193	REG4	150.61	1.29	0.32	3.97	7.16E-05	3.03E-03
ENSG00000171246	NPTX1	148.38	1.63	0.49	3.34	8.45E-04	1.79E-02
ENSG00000177301	KCNA2	144.78	1.04	0.30	3.43	6.05E-04	1.43E-02
ENSG00000099937	SERPIND1	138.39	1.93	0.33	5.79	6.92E-09	2.04E-06
ENSG00000197182	MIRLET7BHG	138.38	1.07	0.19	5.63	1.84E-08	4.36E-06
ENSG00000171759	PAH	134.69	1.57	0.41	3.84	1.22E-04	4.48E-03
ENSG00000172346	CSDC2	123.82	1.09	0.22	4.86	1.15E-06	1.21E-04
ENSG00000280434	AL031595.3	123.77	1.50	0.27	5.61	2.05E-08	4.74E-06
ENSG00000246451	AL049840.1	122.42	1.12	0.21	5.38	7.42E-08	1.33E-05
ENSG00000170271	FAXDC2	121.32	1.35	0.35	3.81	1.37E-04	4.84E-03
ENSG00000126583	PRKCG	117.62	1.06	0.33	3.19	1.43E-03	2.54E-02
ENSG00000100191	SLC5A4	113.70	1.71	0.23	7.30	2.84E-13	3.24E-10
ENSG00000237940	LINC01238	111.82	1.31	0.41	3.20	1.37E-03	2.47E-02
ENSG00000177675	CD163L1	107.40	1.55	0.51	3.05	2.31E-03	3.44E-02
ENSG00000280119	AC093642.2	104.75	1.25	0.41	3.06	2.20E-03	3.34E-02
ENSG00000136542	GALNT5	103.03	1.55	0.49	3.15	1.62E-03	2.76E-02
ENSG00000160307	S100B	100.90	1.57	0.38	4.16	3.21E-05	1.65E-03
ENSG00000232680	AC002511.1	99.76	1.01	0.25	3.98	7.04E-05	2.98E-03
ENSG00000166035	LIPC	99.66	1.07	0.35	3.05	2.33E-03	3.44E-02

Gene stable ID	Gene name	baseMean	log2FoldChange	lfcSE	stat	pvalue	padj
ENSG00000198569	SLC34A3	94.76	1.38	0.39	3.58	3.41E-04	9.27E-03
ENSG00000100146	SOX10	93.66	3.67	1.12	3.26	1.10E-03	2.16E-02
ENSG00000124882	EREG	91.54	4.33	1.01	4.28	1.89E-05	1.09E-03
ENSG00000235688	AC116614.1	86.82	1.07	0.33	3.22	1.28E-03	2.36E-02
ENSG00000137857	DUOX1	86.39	1.45	0.22	6.50	8.05E-11	5.25E-08
ENSG00000066405	CLDN18	83.97	1.42	0.44	3.23	1.23E-03	2.30E-02
ENSG00000154263	ABCA10	83.24	1.00	0.19	5.15	2.60E-07	3.63E-05
ENSG00000279905	AC063926.3	81.78	1.01	0.32	3.18	1.50E-03	2.62E-02
ENSG00000002726	AOC1	73.12	1.44	0.35	4.17	3.02E-05	1.57E-03
ENSG00000188487	INSC	73.09	1.35	0.46	2.94	3.24E-03	4.22E-02
ENSG00000170703	TTLL6	72.81	1.18	0.32	3.73	1.88E-04	6.15E-03
ENSG00000002745	WNT16	71.65	4.84	1.22	3.97	7.32E-05	3.07E-03
ENSG00000259683	AC243562.2	69.80	1.41	0.24	5.83	5.51E-09	1.71E-06
ENSG00000225465	RFPL1S	69.46	1.12	0.34	3.29	1.01E-03	2.02E-02
ENSG00000279716	AC006128.1	68.19	1.32	0.40	3.34	8.42E-04	1.78E-02
ENSG00000104371	DKK4	65.15	1.32	0.40	3.32	9.02E-04	1.87E-02
ENSG00000242268	LINC02082	63.16	1.57	0.43	3.62	2.94E-04	8.44E-03
ENSG00000042062	RIPOR3	60.90	1.49	0.48	3.10	1.95E-03	3.11E-02
ENSG00000167549	CORO6	59.48	1.49	0.41	3.61	3.04E-04	8.64E-03
ENSG00000122711	SPINK4	57.28	1.63	0.55	2.97	2.96E-03	3.99E-02
ENSG00000142484	TM4SF5	54.53	1.86	0.32	5.78	7.26E-09	2.07E-06
ENSG00000168913	ENHO	53.81	1.15	0.32	3.60	3.13E-04	8.78E-03
ENSG00000180998	GPR137C	50.72	1.38	0.28	4.85	1.21E-06	1.25E-04
ENSG00000204347	BTBD17	50.35	1.03	0.35	2.91	3.64E-03	4.56E-02
ENSG00000185897	FFAR3	49.27	1.09	0.30	3.66	2.54E-04	7.63E-03
ENSG00000235890	TSPEAR-AS1	46.65	1.15	0.40	2.89	3.81E-03	4.67E-02
ENSG00000225806	AL121917.1	46.53	1.41	0.24	5.83	5.39E-09	1.70E-06
ENSG00000155659	VSIG4	46.11	1.72	0.55	3.10	1.95E-03	3.11E-02
ENSG00000176884	GRIN1	45.75	1.15	0.33	3.45	5.52E-04	1.33E-02
ENSG00000215018	COL28A1	44.01	1.15	0.30	3.80	1.43E-04	5.01E-03
ENSG00000179270	C2orf71	43.02	1.26	0.34	3.69	2.26E-04	6.99E-03
ENSG00000168306	ACOX2	42.44	1.32	0.43	3.04	2.36E-03	3.47E-02
ENSG00000146250	PRSS35	41.61	2.94	0.54	5.40	6.51E-08	1.22E-05
ENSG00000280334	AC009084.2	39.37	1.27	0.31	4.14	3.44E-05	1.75E-03
ENSG00000281106	TMEM272	38.40	2.83	0.35	8.06	7.45E-16	3.40E-12
ENSG00000256574	OR13A1	38.28	1.01	0.31	3.24	1.20E-03	2.27E-02
ENSG00000102962	CCL22	37.79	3.22	0.77	4.21	2.52E-05	1.37E-03
ENSG00000147588	PMP2	34.49	4.29	0.98	4.36	1.32E-05	8.29E-04
ENSG00000228492	RAB11FIP1P1	34.47	1.05	0.35	2.96	3.08E-03	4.09E-02
ENSG00000160883	HK3	33.89	1.87	0.61	3.08	2.07E-03	3.22E-02
ENSG00000105609	LILRB5	33.79	1.98	0.50	3.93	8.33E-05	3.38E-03
ENSG00000070031	SCT	33.29	2.55	0.56	4.58	4.74E-06	3.81E-04
ENSG00000232504	ST3GAL5-AS1	32.74	1.51	0.41	3.66	2.50E-04	7.53E-03
ENSG00000123560	PLP1	32.14	3.79	0.96	3.94	8.25E-05	3.38E-03
ENSG00000237938	AL450998.3	32.04	1.20	0.42	2.86	4.25E-03	4.99E-02
ENSG00000178773	CPNE7	31.85	1.42	0.37	3.79	1.48E-04	5.12E-03
ENSG00000267629	AC138430.1	31.21	1.92	0.33	5.79	7.16E-09	2.07E-06
ENSG00000149927	DOC2A	30.96	1.29	0.35	3.73	1.91E-04	6.20E-03
ENSG00000226791	AC109826.1	30.85	1.08	0.34	3.14	1.70E-03	2.84E-02
ENSG00000159625	DRC7	30.49	1.16	0.31	3.72	2.02E-04	6.47E-03
ENSG00000172367	PDZD3	29.90	1.93	0.44	4.40	1.06E-05	7.19E-04
ENSG00000163126	ANKRD23	28.70	1.44	0.30	4.87	1.13E-06	1.21E-04
ENSG00000137841	PLCB2	28.21	1.49	0.39	3.80	1.44E-04	5.04E-03
ENSG00000110195	FOLR1	28.17	1.97	0.66	2.98	2.92E-03	3.96E-02
ENSG00000204577	LILRB3	27.85	1.40	0.42	3.35	8.15E-04	1.75E-02
ENSG00000000938	FGR	27.75	2.51	0.87	2.91	3.65E-03	4.56E-02
ENSG00000259668	AC066613.1	26.94	1.14	0.37	3.11	1.90E-03	3.08E-02
ENSG00000178342	KCNG2	26.83	1.65	0.43	3.88	1.06E-04	4.00E-03
ENSG00000145040	UCN2	26.73	1.79	0.62	2.88	3.94E-03	4.78E-02
ENSG00000255035	SDHCP4	26.65	1.50	0.43	3.49	4.92E-04	1.21E-02
ENSG00000227719	AC006042.1	26.49	1.31	0.34	3.84	1.25E-04	4.53E-03
ENSG00000186417	GLDN	26.00	1.88	0.64	2.94	3.24E-03	4.22E-02
ENSG00000174460	ZCCHC12	25.94	1.72	0.55	3.12	1.82E-03	2.99E-02
ENSG00000130711	PRDM12	25.90	1.90	0.62	3.08	2.08E-03	3.23E-02
ENSG00000280332	AC020917.4	25.34	1.13	0.34	3.34	8.37E-04	1.78E-02
ENSG00000213996	TM6SF2	25.28	1.11	0.35	3.16	1.56E-03	2.70E-02
ENSG00000279300	AC008761.3	25.13	1.23	0.40	3.10	1.96E-03	3.11E-02
ENSG00000257242	LINC01619	24.65	1.16	0.38	3.06	2.18E-03	3.33E-02
ENSG00000183691	NOG	24.14	2.86	0.52	5.51	3.58E-08	7.27E-06
ENSG00000117971	CHRNB4	23.33	2.18	0.72	3.02	2.56E-03	3.65E-02
ENSG00000152154	TMEM178A	21.28	1.22	0.35	3.48	5.06E-04	1.24E-02
ENSG00000204121	ECEL1P1	21.09	1.43	0.41	3.47	5.28E-04	1.29E-02
ENSG00000104321	TRPA1	20.60	2.53	0.68	3.72	2.01E-04	6.47E-03
ENSG00000213212	NCLP1	20.57	1.51	0.41	3.67	2.46E-04	7.43E-03
ENSG00000143851	PTPN7	20.53	1.17	0.38	3.09	1.99E-03	3.14E-02
ENSG00000123454	DBH	19.98	1.40	0.47	2.97	3.02E-03	4.04E-02
ENSG00000167895	TMC8	19.44	1.24	0.41	3.05	2.27E-03	3.40E-02
ENSG00000163273	NPPC	19.25	1.52	0.43	3.56	3.78E-04	9.98E-03
ENSG00000109705	NKX3-2	18.87	1.97	0.65	3.02	2.55E-03	3.64E-02
ENSG00000165197	VEGFD	17.76	1.23	0.42	2.91	3.57E-03	4.51E-02
ENSG00000275011	AC129492.6	17.71	2.02	0.63	3.19	1.40E-03	2.51E-02
ENSG00000272797	AC092954.1	17.53	1.75	0.45	3.90	9.61E-05	3.73E-03
ENSG00000197816	CCDC180	17.26	1.56	0.48	3.24	1.21E-03	2.28E-02
ENSG00000254620	AL050327.1	16.90	1.37	0.47	2.91	3.60E-03	4.52E-02
ENSG00000279989	AC011815.3	15.80	1.33	0.43	3.06	2.21E-03	3.35E-02
ENSG00000127364	TAS2R4	15.20	1.33	0.40	3.30	9.81E-04	1.99E-02
ENSG00000100344	PNPLA3	15.04	2.22	0.52	4.30	1.69E-05	1.01E-03
ENSG00000116652	DLEU2L	15.04	1.29	0.42	3.09	2.01E-03	3.17E-02
ENSG00000187140	FOXD3	14.53	3.82	1.12	3.41	6.47E-04	1.50E-02
ENSG00000149534	MS4A2	13.34	1.94	0.60	3.24	1.19E-03	2.26E-02
ENSG00000230417	LINC00595	13.28	1.47	0.49	3.00	2.66E-03	3.74E-02
ENSG00000284738	AL358472.5	13.26	1.33	0.45	3.00	2.73E-03	3.80E-02
ENSG00000259039	AL161804.1	13.14	1.66	0.44	3.75	1.76E-04	5.83E-03
ENSG00000145794	MEGF10	12.21	1.85	0.53	3.50	4.69E-04	1.17E-02

Gene stable ID	Gene name	baseMean	log2FoldChange	lfcSE	stat	pvalue	padj
ENSG00000267577	AC010327.3	12.10	1.51	0.50	3.05	2.27E-03	3.40E-02
ENSG00000146013	GFRA3	12.01	2.75	0.93	2.95	3.16E-03	4.16E-02
ENSG00000142619	PADI3	11.73	2.96	0.87	3.39	6.90E-04	1.57E-02
ENSG00000188869	TMC3	11.55	2.00	0.61	3.29	9.87E-04	1.99E-02
ENSG00000248367	AC008610.1	11.33	1.71	0.48	3.59	3.31E-04	9.08E-03
ENSG00000224713	AC025165.1	11.24	1.60	0.46	3.46	5.47E-04	1.33E-02
ENSG00000223947	AC016738.1	11.13	1.58	0.49	3.25	1.14E-03	2.20E-02
ENSG00000165178	NCF1C	10.97	1.82	0.53	3.46	5.50E-04	1.33E-02
ENSG00000129910	CDH15	10.79	4.05	1.13	3.58	3.47E-04	9.39E-03
ENSG00000279294	AC092135.1	10.52	2.50	0.67	3.71	2.10E-04	6.68E-03
ENSG00000279419	AC004925.1	10.21	1.69	0.57	2.97	3.01E-03	4.04E-02
ENSG00000166104	AC126323.1	10.20	2.08	0.68	3.05	2.29E-03	3.42E-02
ENSG00000171101	SIGLEC17P	10.11	3.45	0.78	4.44	8.81E-06	6.21E-04
ENSG00000226380	AC016831.1	10.06	1.58	0.53	2.96	3.10E-03	4.10E-02
ENSG00000118113	MMP8	9.96	3.18	1.05	3.03	2.46E-03	3.55E-02
ENSG00000243499	RPS6P21	9.88	2.52	0.75	3.38	7.27E-04	1.62E-02
ENSG00000250644	AC068580.4	9.63	1.96	0.66	2.97	2.96E-03	4.00E-02
ENSG00000272564	AC012511.1	9.57	1.87	0.56	3.36	7.78E-04	1.70E-02
ENSG00000182508	LHFPL1	9.47	1.85	0.59	3.15	1.63E-03	2.76E-02
ENSG00000265962	GACAT2	9.10	1.77	0.55	3.21	1.35E-03	2.44E-02
ENSG00000255837	TAS2R20	8.47	1.89	0.66	2.87	4.04E-03	4.85E-02

B) Differentially expressed genes reduced in pseudoislets when compared to islets (>2-fold, padj<0.05).

Gene stable ID	Gene name	baseMean	log2FoldChange	lfcSE	stat	pvalue	padj
ENSG00000184009	ACTG1	40021.50	-1.14	0.26	-4.40	1.08E-05	7.27E-04
ENSG00000100345	MYH9	20638.48	-1.03	0.21	-4.90	9.67E-07	1.07E-04
ENSG00000167552	TUBA1A	17542.41	-1.02	0.28	-3.71	2.11E-04	6.68E-03
ENSG00000115386	REG1A	13255.76	-4.36	1.07	-4.09	4.36E-05	2.06E-03
ENSG00000196924	FLNA	10398.31	-1.07	0.35	-3.02	2.53E-03	3.63E-02
ENSG00000187498	COL4A1	10087.86	-1.30	0.36	-3.62	2.99E-04	8.53E-03
ENSG00000275896	PRSS2	8384.77	-3.86	1.09	-3.54	4.04E-04	1.05E-02
ENSG00000196230	TUBB	8055.82	-1.20	0.17	-7.05	1.80E-12	1.64E-09
ENSG00000111669	TPI1	7693.80	-1.09	0.24	-4.60	4.25E-06	3.46E-04
ENSG00000198959	TGM2	7198.19	-2.09	0.43	-4.85	1.26E-06	1.28E-04
ENSG00000152952	PLOD2	7135.74	-1.53	0.35	-4.39	1.12E-05	7.35E-04
ENSG00000172023	REG1B	6652.22	-8.03	1.10	-7.27	3.49E-13	3.75E-10
ENSG00000129757	CDKN1C	5961.10	-1.06	0.21	-4.95	7.58E-07	8.93E-05
ENSG00000134333	LDHA	5530.08	-2.05	0.39	-5.20	1.96E-07	2.88E-05
ENSG00000058085	LAMC2	5127.70	-1.86	0.51	-3.63	2.86E-04	8.26E-03
ENSG00000171992	SYNPO	5058.94	-1.01	0.27	-3.77	1.66E-04	5.56E-03
ENSG00000147065	MSN	4302.44	-1.06	0.29	-3.60	3.19E-04	8.91E-03
ENSG00000204983	PRSS1	4019.76	-4.41	1.12	-3.93	8.53E-05	3.41E-03
ENSG00000188229	TUBB4B	3664.29	-1.10	0.19	-5.86	4.55E-09	1.48E-06
ENSG00000106366	SERPINE1	3625.73	-1.50	0.48	-3.11	1.87E-03	3.04E-02
ENSG00000171223	JUNB	3338.35	-1.18	0.29	-4.13	3.66E-05	1.83E-03
ENSG00000184489	PTP4A3	3232.54	-1.93	0.37	-5.18	2.19E-07	3.12E-05
ENSG00000091704	CPA1	3112.43	-5.40	1.26	-4.29	1.76E-05	1.04E-03
ENSG00000164266	SPINK1	2900.40	-2.28	0.51	-4.47	7.79E-06	5.62E-04
ENSG00000197930	ERO1A	2895.11	-1.01	0.32	-3.13	1.74E-03	2.90E-02
ENSG00000142789	CELA3A	2894.08	-6.37	1.61	-3.95	7.86E-05	3.25E-03
ENSG00000137331	IER3	2782.01	-1.08	0.30	-3.56	3.70E-04	9.85E-03
ENSG00000116690	PRG4	2762.13	-1.44	0.41	-3.50	4.58E-04	1.15E-02
ENSG00000075240	GRAMD4	2536.98	-1.37	0.27	-5.04	4.73E-07	5.95E-05
ENSG00000134107	BHLHE40	2506.51	-1.42	0.25	-5.60	2.14E-08	4.89E-06
ENSG00000168928	CTRB2	2308.21	-5.24	1.06	-4.93	8.38E-07	9.56E-05
ENSG00000130300	PLVAP	2247.27	-2.18	0.46	-4.71	2.47E-06	2.16E-04
ENSG00000131016	AKAP12	2204.31	-1.05	0.27	-3.93	8.44E-05	3.39E-03
ENSG00000178726	THBD	1877.19	-1.01	0.23	-4.48	7.47E-06	5.46E-04
ENSG00000122884	P4HA1	1811.45	-1.16	0.24	-4.86	1.16E-06	1.21E-04
ENSG00000185043	CIB1	1781.03	-1.01	0.28	-3.61	3.10E-04	8.78E-03
ENSG00000175535	PNLIP	1687.06	-3.77	0.85	-4.41	1.04E-05	7.05E-04
ENSG00000159167	STC1	1676.70	-1.63	0.54	-3.01	2.60E-03	3.69E-02
ENSG00000100906	NFKBIA	1507.93	-1.56	0.46	-3.39	6.94E-04	1.57E-02
ENSG00000153002	CPB1	1489.95	-4.22	0.79	-5.35	8.98E-08	1.53E-05
ENSG00000125148	MT2A	1452.02	-2.59	0.74	-3.50	4.64E-04	1.16E-02
ENSG00000120738	EGR1	1425.32	-2.13	0.49	-4.37	1.21E-05	7.86E-04
ENSG00000164949	GEM	1413.81	-1.23	0.27	-4.48	7.32E-06	5.37E-04
ENSG00000071282	LMCD1	1413.29	-1.22	0.26	-4.70	2.55E-06	2.21E-04
ENSG00000112769	LAMA4	1323.16	-1.19	0.29	-4.09	4.37E-05	2.06E-03
ENSG00000125810	CD93	1266.99	-2.95	0.39	-7.53	4.91E-14	1.01E-10
ENSG00000128567	PODXL	1235.44	-2.49	0.34	-7.40	1.41E-13	1.96E-10
ENSG00000102755	FLT1	1227.91	-2.46	0.43	-5.76	8.60E-09	2.38E-06
ENSG00000204054	LINC00963	1179.78	-1.16	0.32	-3.58	3.48E-04	9.40E-03
ENSG00000157557	ETS2	1166.26	-1.29	0.38	-3.42	6.38E-04	1.49E-02
ENSG00000101384	JAG1	1152.42	-1.33	0.33	-3.98	6.98E-05	2.97E-03
ENSG00000261371	PECAM1	1101.33	-1.76	0.39	-4.51	6.40E-06	4.89E-04
ENSG00000138161	CUZD1	1055.20	-5.24	1.10	-4.75	2.04E-06	1.87E-04
ENSG00000158516	CPA2	1050.84	-4.08	0.98	-4.15	3.28E-05	1.68E-03
ENSG00000170835	CEL	1032.14	-4.71	1.11	-4.25	2.09E-05	1.17E-03
ENSG00000162849	KIF26B	969.67	-1.08	0.24	-4.55	5.44E-06	4.29E-04
ENSG00000137392	CLPS	966.98	-9.17	1.98	-4.63	3.69E-06	3.11E-04
ENSG00000170890	PLA2G1B	962.00	-5.84	1.15	-5.09	3.64E-07	4.85E-05
ENSG00000128342	LIF	896.68	-1.85	0.55	-3.37	7.42E-04	1.64E-02
ENSG00000165434	PGM2L1	889.61	-1.13	0.31	-3.62	2.97E-04	8.49E-03
ENSG00000099860	GADD45B	860.77	-1.84	0.45	-4.06	4.83E-05	2.23E-03
ENSG00000115107	STEAP3	839.81	-2.16	0.33	-6.45	1.11E-10	6.36E-08
ENSG00000162438	CTRC	836.53	-6.20	1.27	-4.86	1.15E-06	1.21E-04
ENSG00000152661	GJA1	816.60	-1.04	0.29	-3.56	3.65E-04	9.77E-03
ENSG00000163909	HEYL	816.03	-1.45	0.38	-3.86	1.12E-04	4.17E-03
ENSG00000110723	EXPH5	802.56	-1.02	0.20	-5.13	2.89E-07	3.94E-05
ENSG00000219073	CELA3B	777.79	-7.63	1.45	-5.28	1.28E-07	2.07E-05
ENSG00000060138	YBX3	768.18	-1.39	0.43	-3.21	1.34E-03	2.44E-02
ENSG00000113916	BCL6	741.50	-1.04	0.28	-3.71	2.04E-04	6.53E-03
ENSG00000128052	KDR	741.32	-2.57	0.31	-8.24	1.75E-16	1.07E-12
ENSG00000176014	TUBB6	737.64	-1.84	0.30	-6.18	6.30E-10	2.74E-07
ENSG00000008517	IL32	712.98	-1.72	0.49	-3.49	4.77E-04	1.18E-02
ENSG00000168209	DDIT4	712.93	-1.10	0.33	-3.30	9.65E-04	1.97E-02
ENSG00000184557	SOCS3	697.44	-2.33	0.45	-5.21	1.85E-07	2.79E-05
ENSG00000158859	ADAMTS4	693.92	-1.77	0.39	-4.59	4.40E-06	3.55E-04
ENSG00000172016	REG3A	681.21	-2.90	0.96	-3.03	2.43E-03	3.54E-02
ENSG00000110799	VWF	660.59	-2.14	0.54	-3.99	6.71E-05	2.89E-03
ENSG00000105974	CAV1	658.54	-1.46	0.38	-3.82	1.35E-04	4.80E-03
ENSG00000168925	CTRB1	654.55	-5.01	1.29	-3.88	1.07E-04	4.00E-03
ENSG00000169715	MT1E	628.79	-2.91	0.72	-4.02	5.83E-05	2.58E-03
ENSG00000111145	ELK3	602.11	-1.18	0.35	-3.41	6.39E-04	1.49E-02
ENSG00000090339	ICAM1	581.21	-1.24	0.42	-2.93	3.43E-03	4.37E-02
ENSG00000151617	EDNRA	573.80	-1.31	0.21	-6.19	6.02E-10	2.68E-07
ENSG00000118515	SGK1	570.85	-1.18	0.29	-4.11	3.92E-05	1.91E-03
ENSG00000069122	ADGRF5	553.78	-2.05	0.35	-5.94	2.82E-09	1.01E-06
ENSG00000154734	ADAMTS1	551.86	-1.79	0.53	-3.38	7.38E-04	1.64E-02
ENSG00000167772	ANGPTL4	544.55	-2.06	0.36	-5.67	1.39E-08	3.51E-06
ENSG00000164283	ESM1	526.31	-2.84	0.44	-6.43	1.26E-10	6.99E-08
ENSG00000127533	F2RL3	511.39	-1.80	0.44	-4.04	5.29E-05	2.38E-03

Gene stable ID	Gene name	baseMean	log2FoldChange	lfcSE	stat	pvalue	padj
ENSG00000187021	PNLIPRP1	496.62	-4.42	1.02	-4.32	1.59E-05	9.61E-04
ENSG00000141639	MAPK4	473.89	-1.15	0.32	-3.64	2.77E-04	8.08E-03
ENSG00000025708	TYMP	473.32	-1.02	0.22	-4.71	2.42E-06	2.14E-04
ENSG00000175197	DDIT3	472.28	-1.13	0.38	-2.93	3.37E-03	4.32E-02
ENSG00000185022	MAFF	469.69	-1.16	0.38	-3.00	2.67E-03	3.74E-02
ENSG00000171564	FGB	468.25	-2.74	0.46	-5.92	3.19E-09	1.12E-06
ENSG00000060982	BCAT1	453.29	-1.42	0.21	-6.63	3.35E-11	2.35E-08
ENSG00000162407	PLPP3	449.81	-1.49	0.26	-5.72	1.07E-08	2.83E-06
ENSG00000221869	CEBPD	439.08	-1.85	0.25	-7.43	1.12E-13	1.70E-10
ENSG00000185567	AHNAK2	437.85	-1.11	0.15	-7.53	5.00E-14	1.01E-10
ENSG00000100311	PDGFB	432.98	-1.62	0.23	-7.02	2.20E-12	1.83E-09
ENSG00000173110	HSPA6	428.50	-4.47	0.84	-5.30	1.14E-07	1.92E-05
ENSG00000069188	SDK2	411.19	-1.07	0.16	-6.76	1.42E-11	1.04E-08
ENSG00000180198	RCC1	409.57	-1.10	0.25	-4.35	1.36E-05	8.46E-04
ENSG00000198417	MT1F	407.52	-2.05	0.54	-3.78	1.58E-04	5.40E-03
ENSG00000139567	ACVRL1	403.65	-1.84	0.37	-4.93	8.07E-07	9.39E-05
ENSG00000137101	CD72	401.37	-1.01	0.20	-5.13	2.83E-07	3.92E-05
ENSG00000176473	WDR25	398.78	-1.15	0.26	-4.39	1.12E-05	7.35E-04
ENSG00000148926	ADM	388.85	-1.58	0.45	-3.50	4.57E-04	1.15E-02
ENSG00000187193	MT1X	387.42	-2.51	0.56	-4.48	7.53E-06	5.48E-04
ENSG00000143954	REG3G	378.79	-4.95	1.72	-2.89	3.91E-03	4.74E-02
ENSG00000057657	PRDM1	349.81	-1.20	0.32	-3.70	2.12E-04	6.69E-03
ENSG00000125144	MT1G	349.64	-4.13	0.85	-4.85	1.25E-06	1.27E-04
ENSG00000172602	RND1	348.45	-1.46	0.40	-3.65	2.61E-04	7.76E-03
ENSG00000112936	C7	347.92	-3.78	0.94	-4.03	5.53E-05	2.46E-03
ENSG00000091879	ANGPT2	313.27	-2.60	0.26	-10.08	6.82E-24	6.23E-20
ENSG00000064666	CNN2	306.39	-1.09	0.34	-3.16	1.56E-03	2.70E-02
ENSG00000137558	PI15	297.03	-3.06	0.47	-6.48	9.20E-11	5.61E-08
ENSG00000010438	PRSS3	294.11	-1.51	0.42	-3.59	3.25E-04	8.98E-03
ENSG00000162618	ADGRL4	291.06	-2.38	0.42	-5.72	1.06E-08	2.83E-06
ENSG00000149564	ESAM	284.56	-2.09	0.40	-5.27	1.40E-07	2.18E-05
ENSG00000152689	RASGRP3	276.28	-1.58	0.33	-4.86	1.15E-06	1.21E-04
ENSG00000001617	SEMA3F	275.55	-1.47	0.35	-4.24	2.19E-05	1.22E-03
ENSG00000185633	NDUFA4L2	275.08	-2.41	0.32	-7.44	1.02E-13	1.69E-10
ENSG00000204301	NOTCH4	267.40	-1.77	0.41	-4.28	1.85E-05	1.07E-03
ENSG00000131634	TMEM204	264.99	-1.14	0.35	-3.27	1.09E-03	2.14E-02
ENSG00000136997	MYC	244.94	-2.18	0.49	-4.49	7.11E-06	5.24E-04
ENSG00000184584	TMEM173	244.28	-1.05	0.26	-4.04	5.38E-05	2.41E-03
ENSG00000137496	IL18BP	237.70	-1.22	0.32	-3.82	1.34E-04	4.77E-03
ENSG00000198873	GRK5	237.26	-1.00	0.28	-3.56	3.68E-04	9.81E-03
ENSG00000135842	FAM129A	237.23	-1.13	0.37	-3.05	2.28E-03	3.42E-02
ENSG00000127329	PTPRB	234.27	-2.07	0.50	-4.14	3.52E-05	1.78E-03
ENSG00000119630	PGF	234.22	-1.68	0.27	-6.15	7.52E-10	3.12E-07
ENSG00000173269	MMRN2	232.71	-2.32	0.40	-5.80	6.75E-09	2.02E-06
ENSG00000066056	TIE1	228.30	-2.06	0.45	-4.57	4.81E-06	3.85E-04
ENSG00000167747	C19orf48	225.13	-1.05	0.31	-3.41	6.40E-04	1.49E-02
ENSG00000123689	G0S2	217.16	-1.62	0.41	-3.93	8.52E-05	3.41E-03
ENSG00000171560	FGA	216.83	-2.61	0.60	-4.38	1.19E-05	7.74E-04
ENSG00000185338	SOCS1	215.79	-1.90	0.42	-4.56	5.16E-06	4.12E-04
ENSG00000120318	ARAP3	213.51	-1.06	0.32	-3.32	8.89E-04	1.85E-02
ENSG00000171557	FGG	213.16	-2.95	0.55	-5.36	8.50E-08	1.49E-05
ENSG00000142615	CELA2A	211.61	-6.00	1.24	-4.83	1.37E-06	1.38E-04
ENSG00000154175	ABI3BP	210.88	-1.30	0.29	-4.55	5.31E-06	4.21E-04
ENSG00000162433	AK4	207.62	-1.95	0.36	-5.43	5.71E-08	1.09E-05
ENSG00000135636	DYSF	203.69	-1.72	0.41	-4.17	3.09E-05	1.60E-03
ENSG00000102760	RGCC	200.06	-1.75	0.27	-6.48	9.22E-11	5.61E-08
ENSG00000102802	MEDAG	190.55	-1.52	0.46	-3.28	1.03E-03	2.05E-02
ENSG00000064989	CALCRL	188.29	-1.90	0.34	-5.67	1.41E-08	3.51E-06
ENSG00000266200	PNLIPRP2	186.25	-2.83	0.99	-2.86	4.19E-03	4.94E-02
ENSG00000174640	SLCO2A1	185.68	-1.95	0.40	-4.90	9.37E-07	1.06E-04
ENSG00000091513	TF	184.67	-4.38	0.96	-4.54	5.51E-06	4.31E-04
ENSG00000197279	ZNF165	183.33	-1.46	0.44	-3.30	9.71E-04	1.98E-02
ENSG00000161940	BCL6B	183.07	-2.29	0.48	-4.81	1.49E-06	1.47E-04
ENSG00000061337	LZTS1	183.03	-1.49	0.41	-3.65	2.60E-04	7.74E-03
ENSG00000157554	ERG	179.18	-2.12	0.43	-4.95	7.48E-07	8.87E-05
ENSG00000144476	ACKR3	176.47	-1.67	0.38	-4.38	1.18E-05	7.66E-04
ENSG00000170989	S1PR1	174.92	-1.99	0.47	-4.20	2.66E-05	1.43E-03
ENSG00000113555	PCDH12	172.71	-1.15	0.24	-4.71	2.50E-06	2.17E-04
ENSG00000115602	IL1RL1	167.90	-5.05	1.33	-3.81	1.41E-04	4.96E-03
ENSG00000247095	MIR210HG	165.43	-1.97	0.32	-6.24	4.51E-10	2.17E-07
ENSG00000167748	KLK1	165.06	-5.71	1.05	-5.43	5.51E-08	1.06E-05
ENSG00000179776	CDH5	165.02	-2.23	0.57	-3.91	9.30E-05	3.62E-03
ENSG00000077942	FBLN1	164.64	-1.91	0.34	-5.64	1.75E-08	4.20E-06
ENSG00000138792	ENPEP	163.63	-1.25	0.32	-3.83	1.26E-04	4.57E-03
ENSG00000154133	ROBO4	161.98	-2.64	0.43	-6.20	5.53E-10	2.52E-07
ENSG00000109511	ANXA10	158.76	-1.43	0.49	-2.90	3.72E-03	4.61E-02
ENSG00000197905	TEAD4	158.55	-2.20	0.41	-5.33	9.83E-08	1.66E-05
ENSG00000100979	PLTP	156.42	-1.11	0.29	-3.88	1.04E-04	3.97E-03
ENSG00000101445	PPP1R16B	154.86	-1.71	0.28	-6.09	1.11E-09	4.31E-07
ENSG00000184613	NELL2	152.72	-1.63	0.48	-3.42	6.16E-04	1.45E-02
ENSG00000129521	EGLN3	152.66	-1.58	0.41	-3.87	1.10E-04	4.09E-03
ENSG00000074660	SCARF1	152.35	-1.36	0.28	-4.88	1.05E-06	1.14E-04
ENSG00000273038	AL365203.2	149.39	-1.26	0.40	-3.17	1.51E-03	2.63E-02
ENSG00000179751	SYCN	147.71	-6.70	1.47	-4.55	5.45E-06	4.29E-04
ENSG00000104332	SFRP1	146.20	-2.63	0.59	-4.47	7.78E-06	5.62E-04
ENSG00000217801	AL390719.1	145.10	-1.26	0.38	-3.35	8.08E-04	1.75E-02
ENSG00000244067	GSTA2	144.43	-2.04	0.56	-3.67	2.43E-04	7.40E-03
ENSG00000183386	FHL3	142.49	-1.22	0.35	-3.51	4.50E-04	1.14E-02
ENSG00000037280	FLT4	139.30	-2.16	0.49	-4.42	9.84E-06	6.73E-04

Gene stable ID	Gene name	baseMean	log2FoldChange	lfcSE	stat	pvalue	padj
ENSG00000107562	CXCL12	138.66	-1.80	0.42	-4.33	1.49E-05	9.16E-04
ENSG00000141540	TTYH2	138.30	-1.01	0.33	-3.02	2.53E-03	3.63E-02
ENSG00000162654	GBP4	137.10	-1.33	0.43	-3.12	1.79E-03	2.96E-02
ENSG00000125740	FOSB	136.75	-2.98	0.66	-4.50	6.93E-06	5.14E-04
ENSG00000167244	IGF2	136.67	-2.15	0.31	-7.00	2.53E-12	2.01E-09
ENSG00000103710	RASL12	134.91	-1.96	0.36	-5.40	6.83E-08	1.25E-05
ENSG00000154783	FGD5	131.39	-1.33	0.42	-3.16	1.58E-03	2.71E-02
ENSG00000164330	EBF1	130.58	-1.02	0.30	-3.35	7.97E-04	1.73E-02
ENSG00000077348	EXOSC5	130.29	-1.24	0.28	-4.37	1.25E-05	7.97E-04
ENSG00000157510	AFAP1L1	130.13	-1.58	0.37	-4.26	2.02E-05	1.14E-03
ENSG00000185615	PDIA2	128.04	-2.74	0.92	-2.98	2.91E-03	3.96E-02
ENSG00000116014	KISS1R	124.90	-1.49	0.40	-3.69	2.24E-04	6.96E-03
ENSG00000177464	GPR4	124.30	-2.39	0.39	-6.10	1.06E-09	4.20E-07
ENSG00000116852	KIF21B	123.17	-1.06	0.30	-3.56	3.78E-04	9.98E-03
ENSG00000017483	SLC38A5	122.02	-2.98	0.61	-4.89	9.96E-07	1.10E-04
ENSG00000104760	FGL1	121.93	-1.72	0.48	-3.60	3.22E-04	8.96E-03
ENSG00000165379	LRFN5	115.79	-1.06	0.28	-3.73	1.91E-04	6.20E-03
ENSG00000184156	KCNQ3	113.25	-1.49	0.40	-3.76	1.71E-04	5.71E-03
ENSG00000113763	UNC5A	111.05	-1.05	0.28	-3.75	1.79E-04	5.89E-03
ENSG00000283632	EXOC3L2	111.01	-2.23	0.38	-5.91	3.43E-09	1.18E-06
ENSG00000107159	CA9	109.71	-3.73	1.20	-3.10	1.95E-03	3.11E-02
ENSG00000105538	RASIP1	100.17	-1.65	0.31	-5.27	1.40E-07	2.18E-05
ENSG00000184497	TMEM255B	99.45	-1.60	0.34	-4.65	3.35E-06	2.84E-04
ENSG00000171388	APLN	98.01	-2.36	0.51	-4.61	3.98E-06	3.32E-04
ENSG00000176435	CLEC14A	97.05	-2.19	0.49	-4.45	8.53E-06	6.06E-04
ENSG00000198844	ARHGEF15	92.65	-1.35	0.45	-2.98	2.85E-03	3.90E-02
ENSG00000120156	TEK	91.56	-2.20	0.40	-5.45	5.08E-08	9.97E-06
ENSG00000164867	NOS3	91.42	-1.53	0.41	-3.69	2.21E-04	6.90E-03
ENSG00000204161	TMEM273	90.93	-1.43	0.29	-4.87	1.12E-06	1.20E-04
ENSG00000133561	GIMAP6	90.43	-1.84	0.54	-3.43	6.00E-04	1.43E-02
ENSG00000237372	AL606807.1	89.87	-2.53	0.32	-7.79	6.74E-15	1.76E-11
ENSG00000003137	CYP26B1	87.74	-1.61	0.36	-4.42	9.69E-06	6.70E-04
ENSG00000230882	AC005077.4	86.02	-1.07	0.27	-3.96	7.56E-05	3.16E-03
ENSG00000124731	TREM1	86.02	-3.01	0.75	-4.00	6.44E-05	2.79E-03
ENSG00000170323	FABP4	82.64	-1.98	0.65	-3.06	2.24E-03	3.38E-02
ENSG00000119919	NKX2-3	82.30	-1.61	0.50	-3.24	1.18E-03	2.25E-02
ENSG00000166148	AVPR1A	81.46	-1.88	0.36	-5.21	1.89E-07	2.81E-05
ENSG00000211448	DIO2	81.45	-1.77	0.39	-4.51	6.38E-06	4.89E-04
ENSG00000095587	TLL2	80.31	-1.93	0.33	-5.89	3.97E-09	1.32E-06
ENSG00000163132	MSX1	79.59	-1.06	0.37	-2.88	4.03E-03	4.84E-02
ENSG00000136630	HLX	79.45	-1.37	0.24	-5.67	1.42E-08	3.51E-06
ENSG00000130755	GMFG	76.82	-1.19	0.32	-3.77	1.65E-04	5.56E-03
ENSG00000171208	NETO2	74.71	-1.19	0.33	-3.64	2.69E-04	7.94E-03
ENSG00000160111	CPAMD8	73.58	-1.40	0.45	-3.10	1.96E-03	3.11E-02
ENSG00000114771	AADAC	73.28	-4.03	1.04	-3.87	1.07E-04	4.00E-03
ENSG00000137672	TRPC6	72.42	-1.93	0.26	-7.37	1.65E-13	2.00E-10
ENSG00000166558	SLC38A8	70.56	-1.87	0.47	-4.00	6.34E-05	2.77E-03
ENSG00000198342	ZNF442	70.40	-1.01	0.28	-3.61	3.05E-04	8.65E-03
ENSG00000179772	FOXS1	69.65	-2.34	0.49	-4.75	2.08E-06	1.87E-04
ENSG00000187800	PEAR1	69.24	-1.44	0.38	-3.83	1.26E-04	4.57E-03
ENSG00000054598	FOXC1	68.59	-1.17	0.32	-3.71	2.10E-04	6.68E-03
ENSG00000189377	CXCL17	68.08	-2.97	0.83	-3.59	3.34E-04	9.12E-03
ENSG00000227218	AL157935.1	64.02	-1.00	0.27	-3.71	2.10E-04	6.68E-03
ENSG00000079337	RAPGEF3	62.67	-1.27	0.36	-3.51	4.55E-04	1.15E-02
ENSG00000007908	SELE	62.24	-3.59	0.64	-5.58	2.38E-08	5.11E-06
ENSG00000120279	MYCT1	60.83	-2.37	0.53	-4.44	9.07E-06	6.34E-04
ENSG00000272636	DOC2B	59.99	-1.56	0.37	-4.26	2.01E-05	1.14E-03
ENSG00000169291	SHE	59.80	-1.61	0.38	-4.30	1.68E-05	1.01E-03
ENSG00000254510	AP001107.5	59.80	-2.36	0.65	-3.65	2.66E-04	7.88E-03
ENSG00000171115	GIMAP8	59.78	-1.33	0.44	-3.00	2.70E-03	3.75E-02
ENSG00000002079	MYH16	59.41	-3.70	0.78	-4.75	2.03E-06	1.86E-04
ENSG00000250899	AC125807.2	59.28	-2.35	0.44	-5.39	7.22E-08	1.31E-05
ENSG00000135063	FAM189A2	58.90	-1.50	0.47	-3.19	1.41E-03	2.52E-02
ENSG00000147113	CXorf36	58.88	-1.53	0.50	-3.05	2.27E-03	3.40E-02
ENSG00000144891	AGTR1	56.12	-1.11	0.34	-3.23	1.24E-03	2.31E-02
ENSG00000256546	AC156455.1	56.09	-1.32	0.38	-3.46	5.45E-04	1.32E-02
ENSG00000118004	COLEC11	54.87	-1.27	0.39	-3.23	1.25E-03	2.33E-02
ENSG00000183638	RP1L1	54.56	-2.16	0.49	-4.40	1.10E-05	7.31E-04
ENSG00000229214	LINC00242	53.42	-1.06	0.36	-2.97	3.00E-03	4.02E-02
ENSG00000226415	TPI1P1	52.86	-1.22	0.41	-2.98	2.92E-03	3.96E-02
ENSG00000189058	APOD	52.54	-2.58	0.53	-4.85	1.23E-06	1.26E-04
ENSG00000152207	CYSLTR2	52.02	-1.16	0.40	-2.93	3.36E-03	4.31E-02
ENSG00000215704	CELA2B	51.87	-5.04	0.99	-5.07	3.97E-07	5.22E-05
ENSG00000146205	ANO7	51.57	-1.04	0.24	-4.37	1.22E-05	7.87E-04
ENSG00000136574	GATA4	49.78	-1.11	0.33	-3.36	7.81E-04	1.70E-02
ENSG00000110852	CLEC2B	49.60	-1.53	0.43	-3.53	4.15E-04	1.07E-02
ENSG00000169427	KCNK9	48.52	-1.75	0.41	-4.22	2.43E-05	1.33E-03
ENSG00000171631	P2RY6	48.40	-1.02	0.35	-2.94	3.32E-03	4.28E-02
ENSG00000232396	AC004882.1	47.44	-2.05	0.41	-4.99	5.91E-07	7.35E-05
ENSG00000205364	MT1M	47.26	-3.50	0.76	-4.64	3.52E-06	2.98E-04
ENSG00000010319	SEMA3G	46.68	-2.47	0.37	-6.62	3.56E-11	2.41E-08
ENSG00000151023	ENKUR	46.38	-1.74	0.33	-5.24	1.59E-07	2.44E-05
ENSG00000267107	PCAT19	45.48	-3.07	0.43	-7.11	1.15E-12	1.10E-09
ENSG00000196639	HRH1	44.48	-1.32	0.35	-3.77	1.64E-04	5.54E-03
ENSG00000214110	LDHAP4	44.43	-1.88	0.63	-2.98	2.92E-03	3.96E-02
ENSG00000253669	GASAL1	43.32	-1.72	0.42	-4.12	3.75E-05	1.86E-03
ENSG00000182162	P2RY8	43.17	-2.66	0.37	-7.16	8.07E-13	8.18E-10
ENSG00000129467	ADCY4	42.66	-1.11	0.37	-3.03	2.44E-03	3.55E-02
ENSG00000102924	CBLN1	42.04	-1.73	0.56	-3.10	1.93E-03	3.11E-02

Gene stable ID	Gene name	baseMean	log2FoldChange	lfcSE	stat	pvalue	padj
ENSG00000237989	LINC01679	41.93	-1.20	0.41	-2.92	3.46E-03	4.40E-02
ENSG00000248323	LUCAT1	41.27	-2.07	0.61	-3.39	6.96E-04	1.57E-02
ENSG00000135547	HEY2	41.19	-1.29	0.42	-3.07	2.11E-03	3.26E-02
ENSG00000134817	APLNR	40.67	-3.63	0.74	-4.92	8.62E-07	9.78E-05
ENSG00000273604	EPOP	40.66	-1.21	0.41	-2.92	3.47E-03	4.40E-02
ENSG00000075340	ADD2	40.54	-1.85	0.54	-3.45	5.66E-04	1.36E-02
ENSG00000243480	AMY2A	40.42	-8.75	1.95	-4.49	7.06E-06	5.22E-04
ENSG00000196839	ADA	39.21	-1.36	0.34	-4.04	5.34E-05	2.40E-03
ENSG00000130052	STARD8	38.99	-1.24	0.31	-3.95	7.74E-05	3.21E-03
ENSG00000227630	LINC01132	38.65	-1.73	0.36	-4.77	1.86E-06	1.74E-04
ENSG00000142677	IL22RA1	38.45	-2.12	0.74	-2.86	4.21E-03	4.96E-02
ENSG00000283406	DGCR5	38.29	-1.05	0.34	-3.10	1.96E-03	3.11E-02
ENSG00000280219	AC093908.1	37.81	-1.20	0.39	-3.05	2.26E-03	3.40E-02
ENSG00000124191	TOX2	36.92	-1.20	0.37	-3.23	1.23E-03	2.30E-02
ENSG00000166736	HTR3A	35.83	-2.95	0.82	-3.57	3.55E-04	9.57E-03
ENSG00000138798	EGF	35.38	-1.81	0.37	-4.95	7.42E-07	8.87E-05
ENSG00000233901	LINC01503	35.03	-1.63	0.50	-3.25	1.14E-03	2.20E-02
ENSG00000197520	FAM177B	34.88	-1.45	0.38	-3.81	1.40E-04	4.94E-03
ENSG00000163083	INHBB	34.85	-1.95	0.64	-3.05	2.27E-03	3.40E-02
ENSG00000235831	BHLHE40-AS1	34.24	-1.42	0.37	-3.81	1.39E-04	4.90E-03
ENSG00000205358	MT1H	34.13	-3.88	0.62	-6.22	5.12E-10	2.40E-07
ENSG00000261051	AC107021.2	33.64	-1.46	0.36	-4.02	5.78E-05	2.56E-03
ENSG00000153162	BMP6	33.35	-2.20	0.45	-4.89	1.02E-06	1.11E-04
ENSG00000134323	MYCN	32.18	-1.22	0.37	-3.27	1.09E-03	2.15E-02
ENSG00000271959	AC100803.3	30.89	-1.86	0.55	-3.39	6.98E-04	1.58E-02
ENSG00000225217	HSPA7	30.16	-4.21	0.85	-4.97	6.73E-07	8.19E-05
ENSG00000283154	IQCJ-SCHIP1	30.06	-1.36	0.46	-2.97	3.02E-03	4.04E-02
ENSG00000164743	C8orf48	29.60	-1.45	0.33	-4.40	1.09E-05	7.27E-04
ENSG00000275302	CCL4	29.37	-1.45	0.45	-3.23	1.23E-03	2.30E-02
ENSG00000167178	ISLR2	29.23	-1.19	0.40	-2.97	2.97E-03	4.00E-02
ENSG00000172548	NIPAL4	27.53	-2.34	0.81	-2.90	3.73E-03	4.61E-02
ENSG00000140519	RHCG	27.45	-3.84	0.90	-4.28	1.84E-05	1.07E-03
ENSG00000273802	HIST1H2BG	27.44	-1.30	0.45	-2.90	3.68E-03	4.57E-02
ENSG00000179144	GIMAP7	27.19	-1.90	0.50	-3.78	1.56E-04	5.35E-03
ENSG00000187848	P2RX2	26.97	-2.11	0.66	-3.22	1.29E-03	2.37E-02
ENSG00000189410	SH2D5	26.85	-2.32	0.78	-2.96	3.11E-03	4.11E-02
ENSG00000232192	KIF26B-AS1	26.16	-1.26	0.40	-3.14	1.67E-03	2.81E-02
ENSG00000179674	ARL14	25.70	-2.39	0.76	-3.16	1.57E-03	2.70E-02
ENSG00000168298	HIST1H1E	25.13	-1.39	0.43	-3.22	1.28E-03	2.36E-02
ENSG00000101230	ISM1	24.37	-1.84	0.55	-3.34	8.36E-04	1.78E-02
ENSG00000117245	KIF17	24.37	-1.54	0.36	-4.27	1.99E-05	1.13E-03
ENSG00000175084	DES	24.16	-2.20	0.57	-3.88	1.05E-04	3.99E-03
ENSG00000163673	DCLK3	23.89	-2.07	0.66	-3.16	1.58E-03	2.71E-02
ENSG00000185467	KPNA7	23.59	-3.20	1.10	-2.90	3.77E-03	4.64E-02
ENSG00000114204	SERPINI2	22.99	-4.41	0.93	-4.75	2.07E-06	1.87E-04
ENSG00000284882	AL359762.1	22.60	-1.14	0.35	-3.31	9.33E-04	1.92E-02
ENSG00000176887	SOX11	22.06	-2.04	0.60	-3.38	7.15E-04	1.60E-02
ENSG00000147257	GPC3	21.69	-1.99	0.62	-3.20	1.39E-03	2.49E-02
ENSG00000132321	IQCA1	21.43	-1.31	0.43	-3.03	2.46E-03	3.55E-02
ENSG00000101938	CHRDL1	21.05	-2.41	0.78	-3.09	2.02E-03	3.17E-02
ENSG00000230472	AL135908.1	20.63	-3.08	0.83	-3.73	1.91E-04	6.20E-03
ENSG00000227038	GTF2IP7	20.52	-1.41	0.46	-3.06	2.23E-03	3.36E-02
ENSG00000259466	NPM1P47	20.07	-2.47	0.52	-4.78	1.76E-06	1.67E-04
ENSG00000213085	CFAP45	19.42	-1.43	0.46	-3.10	1.96E-03	3.11E-02
ENSG00000127589	TUBBP1	19.04	-1.54	0.37	-4.11	3.90E-05	1.91E-03
ENSG00000185668	POU3F1	18.96	-1.25	0.43	-2.91	3.57E-03	4.50E-02
ENSG00000280255	AC004947.2	18.35	-1.48	0.44	-3.34	8.24E-04	1.76E-02
ENSG00000224189	HAGLR	18.28	-2.04	0.64	-3.18	1.47E-03	2.60E-02
ENSG00000122679	RAMP3	17.68	-1.89	0.58	-3.26	1.10E-03	2.16E-02
ENSG00000162367	TAL1	17.44	-1.59	0.42	-3.82	1.34E-04	4.77E-03
ENSG00000283265	AL356234.3	16.18	-1.94	0.55	-3.53	4.21E-04	1.08E-02
ENSG00000225285	LINC01770	15.88	-1.65	0.51	-3.26	1.12E-03	2.17E-02
ENSG00000237643	AL365226.2	15.50	-5.29	1.20	-4.42	9.79E-06	6.72E-04
ENSG00000277632	CCL3	15.48	-1.75	0.45	-3.85	1.19E-04	4.36E-03
ENSG00000130307	USHBP1	15.11	-1.90	0.48	-3.92	8.79E-05	3.48E-03
ENSG00000164736	SOX17	14.70	-1.71	0.53	-3.26	1.11E-03	2.16E-02
ENSG00000158683	PKD1L1	14.60	-1.84	0.57	-3.23	1.24E-03	2.31E-02
ENSG00000177398	UMODL1	14.57	-1.55	0.49	-3.19	1.42E-03	2.54E-02
ENSG00000157856	DRC1	14.46	-2.15	0.73	-2.95	3.18E-03	4.17E-02
ENSG00000234292	AC123595.1	14.30	-2.81	0.68	-4.14	3.54E-05	1.78E-03
ENSG00000080709	KCNN2	14.28	-1.95	0.59	-3.33	8.56E-04	1.80E-02
ENSG00000240509	RPL34P18	13.85	-1.25	0.41	-3.06	2.22E-03	3.35E-02
ENSG00000256925	ADGRA1-AS1	13.68	-1.55	0.53	-2.91	3.60E-03	4.52E-02
ENSG00000120149	MSX2	13.63	-2.12	0.70	-3.03	2.45E-03	3.55E-02
ENSG00000249751	ECSCR	12.67	-2.49	0.61	-4.06	4.96E-05	2.26E-03
ENSG00000188596	CFAP54	12.08	-1.47	0.51	-2.88	3.92E-03	4.76E-02
ENSG00000171517	LPAR3	11.68	-4.45	1.19	-3.73	1.93E-04	6.25E-03
ENSG00000153495	TEX29	11.63	-2.23	0.75	-2.97	2.94E-03	3.97E-02
ENSG00000237250	AL359924.1	11.41	-2.30	0.58	-3.97	7.27E-05	3.06E-03
ENSG00000142748	FCN3	11.32	-4.07	0.94	-4.34	1.42E-05	8.82E-04
ENSG00000123685	BATF3	11.22	-1.95	0.52	-3.77	1.66E-04	5.56E-03
ENSG00000236345	AL354719.2	11.16	-2.95	0.93	-3.17	1.52E-03	2.64E-02
ENSG00000162444	RBP7	11.15	-1.74	0.50	-3.50	4.66E-04	1.16E-02
ENSG00000241158	ADAMTS9-AS1	10.45	-5.31	1.31	-4.07	4.68E-05	2.19E-03
ENSG00000146399	TAAR1	10.27	-1.89	0.60	-3.18	1.47E-03	2.60E-02
ENSG00000186564	FOXD2	10.25	-1.50	0.50	-3.02	2.55E-03	3.64E-02
ENSG00000111886	GABRR2	10.15	-1.57	0.50	-3.12	1.78E-03	2.95E-02
ENSG00000150048	CLEC1A	9.98	-2.78	0.73	-3.82	1.33E-04	4.77E-03
ENSG00000263571	AC004147.2	9.77	-1.81	0.55	-3.26	1.10E-03	2.16E-02

Gene stable ID	Gene name	baseMean	log2FoldChange	lfcSE	stat	pvalue	padj
ENSG00000205362	MT1A	9.75	-2.43	0.65	-3.76	1.73E-04	5.74E-03
ENSG00000248771	SMIM31	9.55	-1.88	0.58	-3.21	1.34E-03	2.44E-02
ENSG00000144119	C1QL2	9.46	-4.56	1.32	-3.46	5.32E-04	1.29E-02
ENSG00000054938	CHRDL2	9.36	-2.51	0.78	-3.22	1.28E-03	2.36E-02
ENSG00000145283	SLC10A6	9.30	-3.02	0.74	-4.06	4.84E-05	2.23E-03
ENSG00000284395	AL032819.3	9.29	-2.66	0.78	-3.42	6.22E-04	1.46E-02
ENSG00000231483	AL365356.4	9.13	-2.80	0.83	-3.39	6.96E-04	1.57E-02
ENSG00000267097	SLC14A2-AS1	9.07	-2.61	0.71	-3.68	2.38E-04	7.26E-03
ENSG00000277749	AC023300.2	9.00	-2.14	0.67	-3.22	1.27E-03	2.34E-02
ENSG00000240602	AADACP1	8.45	-2.34	0.71	-3.29	9.88E-04	1.99E-02
ENSG00000178821	TMEM52	8.38	-2.47	0.80	-3.08	2.10E-03	3.25E-02

Supplementary Table 2 Pseudoislet preparations display reduced mRNA levels of exocrine and endothelial cell markers compared to islets. Differentially expressed genes (>2-fold, padj<0.05) between human islets and pseudoislets

	Protein	Gene name	islet Mean	log2fold Change	padj	Expression cells					
						Acinar	Duct	Endo-thelial	PSCs	MII/M	Endo-crine
Acinar and Ductal	Serine Peptidase Inhibitor, Kazal Type 1	SPINK1	2900.4	-2.28	5.62E-04	+++	++				+
	Serine proteases family	PRSS1	4019.8	-4.41	3.41E-03	+++	++				+
		PRSS2	8384.8	-3.86	1.05E-02	++					
		PRSS3	294.1	-1.51	8.98E-03	+++	+				+
		PRSS35	41.6	2.94	1.22E-05		+				
		PRSS23	4668.1	-0.79	4.78E-03	+	++				+
	Colipase	CLPS	967.0	-9.17	3.11E-04	+++	++				
	Chymotrypsin C	CTRC	836.5	-6.20	1.21E-04	+++					
	Carboxypeptidase A	CPA1	3112.4	-5.40	1.04E-03	+++					
		CPA2	1050.8	-4.08	1.68E-03	+++					
	Carboxypeptidase B	CPB1	1490.0	-4.22	1.53E-05	+++	+				+
	Chymotrypsinogen B	CTRB1	654.6	-5.01	4.00E-03	+++	+				
		CTRB2	2308.2	-5.24	9.56E-05	+++	+				
	Chymotrypsin Like Elastase	CELA2A	211.6	-6.00	1.38E-04	+++	+				
		CELA2B	51.9	-5.04	5.22E-05	+++					
		CELA3A	2894.1	-6.37	3.25E-03	+++	+				
		CELA3B	777.8	-7.63	2.07E-05	+++					
	Kallikrein 1	KLK1	165.1	-5.71	1.06E-05	+++					
	Pancreatic Lipase	PNLIP	1687.1	-3.77	7.05E-04	+++					
	Pancreatic Lipase-Related Protein	PNLIPRP1	496.6	-4.42	9.61E-04	+++					
		PNLIPRP2	186.2	-2.83	4.94E-02	+++					
	Regenerating Family Member	REG1A	13255.8	-4.36	2.06E-03	+++	++				+
		REG1B	6652.2	-8.03	3.75E-10	+++	+				
		REG3A	681.2	-2.90	3.54E-02	+++	+				+
		REG3G	378.8	-4.95	4.74E-02	+++					
	Carboxyl Ester Lipase	CEL	1032.1	-4.71	1.17E-03	+++					
	Phospholipase A2 Group IB	PLA2G1B	962.0	-5.84	4.85E-05	+++	+				+
	GATA Binding Protein 4	GATA4	49.8	-1.11	1.70E-02	++	+				
	Syncollin	SYCN	147.7	-6.70	4.29E-04	++					
	Amylase Alpha 2A	AMY2A	40.4	-8.75	5.22E-04	+					
	Serpin Family I Member 2	SERPINI2	23.0	-4.41	1.87E-04	++					
	Arylacetamide Deacetylase	AADAC	73.3	-4.03	4.00E-03	++					
	Metallothionein 1H	MT1H	34.1	-3.88	2.40E-07	+++	++				
Endothelial	Fms Related Tyrosine Kinase 1	FLT1	1227.9	-2.46	2.30E-06			+++			
	Kinase Insert Domain Receptor	KDR	741.3	-2.57	1.07E-12			+++			
	**C**luster of **D**ifferentiation 93	CD93	1267.0	-2.95	1.01E-10			+++			
	Endothelial Cell Adhesion Molecule	ESAM	284.6	-2.09	2.18E-05		+	+++			
	Platelet And Endothelial Cell Adhesion Molecule 1	PECAM1	1101.3	-1.76	4.89E-04			+++			
	Endothelial Cell Specific Molecule 1	ESM1	526.3	-2.84	6.99E-08			+++			
	Solute Carrier Organic Anion Transporter Family Member 2A1	SLCO2A1	185.7	-1.95	1.06E-04	+	+	++			
	Plasmalemma Vesicle Associated Protein	PLVAP	2247.3	-2.18	2.16E-04			+++			
	Regulator Of Cell Cycle	RGCC	200.1	-1.75	5.61E-08			+++			
	SMAD6	SMAD6	226.2	-0.84	1.06E-04	+	+	++			
	erythroblast transformation-specific Transcription Factor	ERG	179.2	-2.12	8.87E-05			+++			
	Beta-Interferon Gene Positive-Regulatory Domain I Binding Factor	PRDM1	349.8	-1.20	6.69E-03			+++	+		
	Transcription Factor 4	TCF4	1717.9	-0.31	2.81E-02			+++			+
	Neurogenic Locus Notch Homolog Protein	NOTCH4	267.4	-1.77	1.07E-03			++			
	NK2 Homeobox 3	NKX2-3	82.3	-1.61	2.25E-02			+			
	ETS Proto-Oncogene 1, Transcription Factor	ETS1	2568.9	-0.83	5.10E-06	+	+	+++	+		+
	Intercellular Adhesion Molecule 1	ICAM1	581.2	-1.24	4.37E-02	+	++	+++			+
	laminin subunit alpha 4	LAMA4	1323.2	-1.19	2.06E-03			+++	++		
	collagen type IV alpha 1 chain	COL4A1	10087.9	-1.30	8.53E-03		+	+++	+++		
	multimerin 2	MMRN2	232.7	-2.32	2.02E-06			++			
	von Willebrand factor	VWF	660.6	-2.14	2.89E-03			++			
	cadherin 5	CDH5	165.0	-2.23	3.62E-03			+++			
	protocadherin 12	PCDH12	172.7	-1.15	2.17E-04			++			
	scavenger receptor class F member 1	SCARF1	152.4	-1.36	1.14E-04		+	++			+
	selectin E	SELE	62.2	-3.59	5.11E-06			+			
